# Water-Based Extraction of Bioactive Principles from Blackcurrant Leaves and *Chrysanthellum americanum*: A Comparative Study

**DOI:** 10.3390/foods9101478

**Published:** 2020-10-16

**Authors:** Phu Cao-Ngoc, Laurent Leclercq, Jean-Christophe Rossi, Jasmine Hertzog, Anne-Sylvie Tixier, Farid Chemat, Rouba Nasreddine, Ghassan Al Hamoui Dit Banni, Reine Nehmé, Philippe Schmitt-Kopplin, Hervé Cottet

**Affiliations:** 1 IBMM, University of Montpellier, CNRS, ENSCM, 34093 Montpellier, France; tech.ngocphu@gmail.com (P.C.-N.); jean-christophe.rossi@umontpellier.fr (J.-C.R.); 2 Analytical BioGeoChemistry, Helmholtz Zentrum Muenchen, 85764 Neuherberg, Germany; jasmine.hertzog@gmail.com (J.H.); schmitt-kopplin@helmholtz-muenchen.de (P.S.-K.); 3 Analytical Food Chemistry, Technische Universität Muenchen, 85354 Freising, Germany; 4 GREEN Extraction Team, INRA, University of Avignon, 84916 Avignon, France; anne-sylvie.fabiano@univ-avignon.fr (A.-S.T.); farid.chemat@univ-avignon.fr (F.C.); 5 Institute of Organic and Analytical Chemistry (ICOA), CNRS, University of Orléans, 45067 Orléans, France; rouba.nasreddine@univ-orleans.fr (R.N.); ghassan.al-hamoui-dit-banni@univ-orleans.fr (G.A.H.D.B.); reine.nehme@univ-orleans.fr (R.N.)

**Keywords:** blackcurrant, hawthorn, Chrysanthellum *americanum*, water-based extraction, polyphenol, flavonoid, procyanidin, granulometry, infusion, enzymatic activity

## Abstract

The water-based extraction of bioactive components from flavonoid-rich medicinal plants is a key step that should be better investigated. This is especially true when dealing with easy-to-use home-made conditions of extractions, which are known to be a bottleneck in the course for a better control and optimization of the daily uptake of active components from medicinal plants. In this work, the water-based extraction of Blackcurrant (*Ribes nigrum*) leaves (BC) and *Chrysanthellum americanum* (CA)*,* known to have complementary pharmacological properties, was studied and compared with a previous work performed on the extraction of Hawthorn (*Crataegus,* HAW). Various extraction modes in water (infusion, percolation, maceration, ultrasounds, microwaves) were compared for the extraction of bioactive principles contained in BC and CA in terms of extraction yield, of amount of flavonoids, phenolic compounds, and proanthocyanidin oligomers, and of UHPLC profiles of the extracted compounds. The qualitative and quantitative aspects of the extraction, in addition to the kinetic of extraction, were studied. The optimized easy-to-use-at-home extraction protocol developed for HAW was found very efficient to easily extract bioactive components from BC and CA plants. UHPLC-ESI-MS and high-resolution Fourier transform ion cyclotron resonance mass spectrometry (FT-ICR MS) were also implemented to get more qualitative information on the specific and common chemical compositions of the three plants (including HAW). Their antihyaluronidase, antioxidant, and antihypertensive activities were also determined and compared, demonstrating similar activities as the reference compound for some of these plants.

## 1. Introduction

The extraction of bioactive principles from medicinal plants depends on a great number of factors, such as the extraction time, the extraction temperature, the granulometry of the dry plant, the relative proportion of plant and solvent used for the extraction, to cite only some of them [1]. To promote herbal medicine and favor its acceptance in modern Western integrative medicine [2] and to meet the increasing societal demand in that field [3], it is crucial to investigate and optimize the extraction protocol, so that a daily uptake of active components can be obtained in a repeatable way.

In a previous study [4] the extraction of Hawthorn (*Crataegus*, abbreviated HAW in the following) in water was thoroughly investigated leading to a simple, fast, and optimized protocol that can be used by anyone at home. If the plant is ground (typically with a granulometry <1 mm using a commercially grinder), infusion with simple manual stirring is the easiest way to extract bioactive components from HAW, and the other extraction modes (maceration, ultrasounds, microwaves, percolation) did not significantly improve the extraction yield. The optimized protocol (3 min infusion of 2.5 g hawthorn ground flowering tops in 250 mL boiling water) with a French-press coffee maker (no infusion bags) can afford a quotidian intake of polyphenols, flavonoids, and proanthocyanidin oligomers similar to the recommended dose issued from standardized hawthorn extracts [4]. Extraction yields about 22% in mass can be reached in a repeatable and controlled way, with a quantified daily uptake of active components.

HAW is used for its cardiotonic, vasodilative, hypotensive, sedative, antihyperlipidemic, and antiatherosclerotic properties. In this work, we aim at pursuing our study initiated with HAW by investigating two other plants of complementary known pharmacological activities, namely blackcurrant leaves (*Ribes nigrum*) (BC) and *Chrysanthellum americanum* (CA). Blackcurrant (*Ribes nigrum*) is a woody shrub that is widely cultivated across temperate Europe, Russia, New Zealand, parts of Asia, and to a lesser extent North America [5,6]. BC contain a valuable source of bioactive compounds especially anthocyanins, proanthocyanidins, phenolic acids, flavonoids, and also vitamin C [7]. Since ancient times, BC have been generally used in European folk medicines to treat arthritis, rheumatism, and respiratory problems [8] due to its antioxidant, anti-inflammatory, and antimicrobial activities, as well as its vasomodulatory, antihemostatic, and muscle-relaxing effects, and even some neuroprotective and cancer-preventive activities [6,9,10,11,12,13,14,15]. *Chrysanthellum americanum*, a genus of yellow flowering plants in the *Chrysanthemum* family [16,17], grows mainly in the mountainous regions or moderate altitude areas in Africa from Senegal to Nigeria and South America from southern Mexico to northern Brazil [18,19]. CA has been traditionally used for its significant wound healing properties in African and American folk medicine and in the treatment of fever, hepatitis, jaundice, and dysentery [20]. In Cuba traditional medicine, it has been used for gastro-intestinal pains, rheumatism, and kidney diseases [19]. More recently, CA was reported for its action against irritable bowel syndrome [21] and for its hepatoprotective properties (mainly evidenced against ethylic alcohol and CCl_4_), lipid lowering actions, and its positive effects against vascular diseases by promoting blood microcirculation [18,19,20,22,23,24,25]. The pharmacological properties of CA are generally attributed to saponosides (such as chrysanthellins A and B) and flavonoids compounds. However, CA was much less studied in the literature compared to HAW and BC. These three medicinal plants were selected in this work for comparison, because their biological activities, which are different one each other, are based on the plant totum, or at least on their flavonoid and proanthocyanidin oligomer contents. 

In this work, we first wanted to demonstrate that the easy-to-use-at-home extraction protocol developed for HAW was also optimized for other medicinal plants (BC, CA). For that purpose, various extraction modes in water (infusion, percolation, maceration, ultrasounds, microwaves) were compared for BC and CA in terms of extraction yield, of amount of flavonoids, phenolic compounds, and proanthocyanidin oligomers in the extracted compounds. The qualitative and quantitative aspects of the extraction, in addition to the kinetic of extraction were studied. The second objective of this work was to investigate how different the chemical composition of these three medicinal plants is. For that, UHPLC profiles and high-resolution Fourier transform ion cyclotron resonance mass spectrometry (FT-ICR MS) were implemented to get more information on the specific and common chemical compositions. Finally, their potential antihyaluronidase, antioxidant, and antihypertensive activities were also determined and compared by a global first-line screening. This allowed us to obtain the percentage of inhibition and antioxidant capacities induced by the different prepared water-based extracts, which is sufficient to compare their bioactivities. Comparison with a control or a reference compound allowed obtaining a precise indication about the effective actions of the extracts.

## 2. Materials and Methods 

### 2.1. Chemicals

Dry *Chrysanthellum americanum* (lot n° 559980, n° CP44120, n° 558088, origin Ivory Coast), dry blackcurrant leaves (*Ribes nigrum*, lot n° 55870, lot n° 558024, origin Poland), and dry hawthorn flowering tops (*Crataegus*, lot n°20335, lot n° CB58120, lot n° APC27031904, origin France) raw materials were purchased from France Herboristerie (Noidans-Lès-Vesoul, France). Analytical grade reagents were used as received without any further purification. Folin–Ciocalteu reagent, aluminum chloride hexahydrate (AlCl_3_.6H_2_O), sodium carbonate (Na_2_CO_3_), n-butanol (CH_3_-(CH_2_)_3_-OH), methanol (CH_3_OH), ammonium iron(III) sulfate dodecahydrate (NH_4_Fe(SO_4_)_2_.12 H_2_O), hydrochloric acid (HCl), quercetin (Q), gallic acid (GA), cyanidin chloride (CY), ammonium acetate (CH_3_COONH_4_, purity ≥98%), epigallocatechin gallate (EGCG, purity ≥95%), hyaluronidase type I-S from bovine testes (BTH, 400–1000 units mg^−1^ solid), sodium acetate (CH_3_COONa, purity ≥99%), sodium hydroxide (NaOH, purity ≥98%) oligohyaluronic acid (oligo-HA4 or tetrasaccharide (Tet), C_28_H_44_N_2_O_23_), and trolox (purity ≥98%) were purchased from Merck (Saint-Quentin Fallavier, France). Hyaluronic acid, sodium salt, *Streptococcus pyrogenes* (HA, from Calbiochem) were purchased from Merck Millipore (Molsheim, France). ACE kit-WST was purchased from Dojindo Laboratories (Kumamoto, Japan). 2,2′-azino-bis-(3-ethylbenzathiazoline-6-sulfonic acid) (ABTS, purity = 98%) reagent was purchased from ThermoFisher-Alfa Aesar (Kandel, Germany). Ammonia (NH_4_OH, 28%) and glacial acetic acid (CH_3_COOH) were purchased from VWR International (Fontenay-sous-Bois, France). Syringes and hydrophilic polyvinylidenedifluoride (PVDF) Econo Syringe Filters, with pore size 0.2 μm, were purchased from Agilent, USA. Ultrapure water was obtained with a MilliQ system from Millipore (Molsheim, France). 

### 2.2. Ground Plant, Granulometry and Density

Three different grinders were used to grind the dry plants. KG79 Delonghi (Trevise, Italy) grinder was used at the two positions named ‘coarse’ and ‘fine’ to obtain ‘coarse’ and ‘fine’ plant materials, respectively. MKM6003 Bosch grinder (Munich, Germany) was used at two manual shaking times (10 and 30 s) to get ‘ultrafine 10 s’ and ‘ultrafine 30 s’ plant materials, respectively. MF10 basic laboratory Ika grinder (Ika-Werke GmbH, Staufen, Germany) was used to obtain ‘1 mm’ plant materials. Plant material density was basically determined by measuring the volume of 2 g plant material in a 10 mL (or 25 mL) graduated test tube (in triplicate). Dry laser Malvern Panalytical granulometer (Royston, United Kingdom) was used to determine the distribution in size of each plant material. 

### 2.3. Infusion Extraction

A 500 mL three-necked flask with an olive magnetic stirrer was used for infusion extractions [4], in which 2.5 g of dry plant was placed and 250 mL boiling water was added. The mixture was stirred at 500 rpm. An Ebro EBI20-IF temperature sensor (Ingolstadt, Germany) was used to follow the temperature decrease upon time. Each experiment was performed in triplicate.

### 2.4. Maceration Extraction

A 500 mL three-necked flask with an olive magnetic stirrer, an oil bath, and a FB15002 heating magnetic stirrer equipped with a digital thermo-regulator (Fisher Scientific, Illkirch, France) were used for maceration extractions [4]. In total, 2.5 g of dry plant and 250 mL water were added. The mixture was stirred at 500 rpm and the temperature was set at 60 °C. Each experiment was performed in triplicate.

### 2.5. Ultrasound-Assisted Extraction

A UIP 1000 hdT ultrasonic homogenizer with 1 kW power (Hielscher Ultrasonics GmbH, Teltow, Germany) was used for ultrasound-assisted (US) extractions [4]. A 1 L double jacket reactor equipped with a mechanical stirrer (IKA RSC classic, Germany) and a temperature sensor, was used to carry out the experiments. A cooling system was used to maintain constant the temperature. In total, 2.5 g of dry plant and 250 mL water were added. The mixture was mechanically stirred at 250 rpm and the temperature was set at 60 °C. Each experiment was performed in triplicate.

### 2.6. Microwave-Assisted Extraction

A NEOS-GR mono-mode microwave apparatus with a closed-vessel system (Milestone Sarl, Sorisole, Italy) was used for microwave-assisted (MW) extractions [4]. A 500 mL flask containing 2.5 g of dry plant and 250 mL water, was placed in the MW oven. The power was set at 300 W. No stirring was applied. Under these experimental conditions, temperature rapidly increased reaching 95 °C in 10 min. Each experiment was performed in triplicate.

### 2.7. Percolation Extraction

A KRUPS coffee percolator (Solingen, Germany) was used for percolation extractions [4]. In total, 2.5 g of dry plant was put in a filter and 250 mL water was used. No stirring was applied. Temperature rapidly increased reaching 100 °C after a few seconds. The extracted solution obtained after 5 min percolation was passed again in the percolator for 5 min more. Each experiment was performed in triplicate.

### 2.8. Optimized Easy-To-Use Infusion Extraction

A ‘French press’ (Bodum^®^, Bistro model, Triengen, Switzerland) was used for the infusion of 2.5 g ground material (see Section 2.2) in 250 mL boiling water [4]. Manual rotation of the recipient was sufficient to ensure good initial mixing of the plant in the boiling water, i.e., magnetic stirring was not required to get optimal extraction from ground plant. After 10 min extraction, the herbal tea solution was filtrated first with the Bodum^®^ cover to eliminate the largest solid plant particles, then with Whatman filter paper. The herbal tea solution was finally concentrated and lyophilized to get dry extract.

### 2.9. Kinetic Monitoring

**Figure 1:**
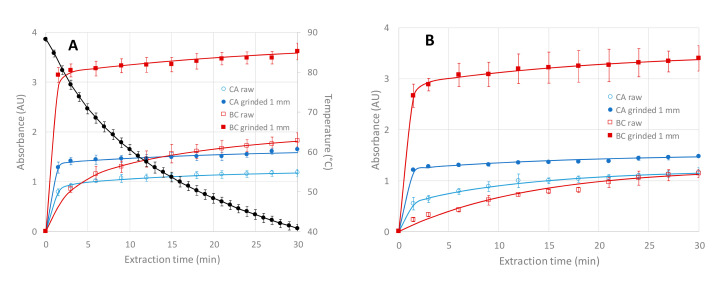
Kinetics of extraction of *Chrysanthellum americanum* (CA, lot no. 559980) and blackcurrant (BC, lot no. 55870) monitored by UV absorbance at 198 nm. (**A**) Infusion mode at 500 rpm stirring speed with the temperature profile (in black). (**B**) Maceration mode at 500 rpm stirring speed and at 60 °C. A total of 2.5 g of plant material in 250 mL water was systematically used. In total, 100 μL of the solution was taken and added to 4 mL water before each UV measurement. If the absorbance values were above 1.7, dilution in 8 mL water (instead of 4 mL) was performed, but the experimental values were multiplied by 2 to allow for a comparison with dilutions in 4 mL. Error bars are ± 1 SD on *n* = 3 repetitions of independent extractions. For ground materials, 1 mm grinder was used (see Section 2.2).

UV spectrophotometer (Perkin-Elmer model Lambda 20, Wellesley, MA, USA) was used to monitor the kinetics of extraction. UV absorbance at 198 nm was determined using 1 mL quartz cells (Hellma GmbH, Müllheim, Germany). Then, 100 µL of herbal tea solution was taken and added to 4 mL water. If the absorbance values were above 1.7 AU, 4 mL more water was added. UV measurement was realized after shortly vortex-mixing. In total, 100 µL fresh water was added into the three-necked flask reactor to keep constant the total volume. A total of 100 µL water instead of herbal tea solution was used to set zero absorbance value. The absorbance *A*(*t*) vs. extraction time *t* kinetic curves were fitted according to Equation (1) using Excel solver (see plain lines in Figure 1A):

(1) $$A(t)=A_{\infty }-A_{1}\exp(-\frac{t}{\tau_{1}})-\left(A_{\infty }-A_{1}\right)\exp(-\frac{t}{\tau_{2}})$$

where *τ*_1_ and *τ*_2_ are two characteristic extraction times, *A*_1_ is a fitting parameter corresponding to an intermediate extraction plateau, and $A_{\infty }$ is the maximum absorbance at infinite extraction time.

### 2.10. Preparation of the Extracts and Global Extraction Yields

The herbal tea solution issued from each extraction protocol (including the solid plant residues) was first filtrated with a Whatman filter paper put on a Büchner funnel and a KNF N820FT.18 vacuum pump (Freiburg, Germany). A rotary evaporator was used to concentrate the extract until 10 mL volume and the resulting solution was finally lyophilized (CRIOS-80 Cryotec, Saint-Gély-du-Fesc, France). Freeze-dried extracts were weighed for the calculation of the global extraction yield expressed in mass % of solid extract compared to the dry plant initial mass. All the solid extracts were stored at 4 °C.

### 2.11. Total Polyphenols Content (TPC)

The Folin–Ciocalteu method was used to assess the total polyphenols content (TPC) in plant extracts, as described by Singleton and Rossi [26]. In total, 100 µL of plant extract solution (at 20 mg mL^−1^ in water) was added to 3 mL water and 200 µL Folin–Ciocalteu reagent. After 3 min, 1 mL of 20% sodium carbonate aqueous solution was added. After vortexing for 2 min and incubating in darkness at RT for 90 min, the resulting solution was then centrifuged for 3 min at 8000 rpm (Sigma Model 302 K, Osterode am Harz, Germany). The absorbance was measured at 760 nm (see Section 2.9 for the equipment). Standard calibration curve was realized with GA (0–250 mg L^−1^). The results were expressed as mg GA equivalent per gram of dry plant and calculated as mean value ± 1 SD (*n* = 3). Herbal tea solution was replaced by water to set zero absorbance value.

### 2.12. Total Flavonoids Content (TFC)

Aluminum chloride method was used to assess the total flavonoids content (TFC) in plant extracts, as described by Lamaison and Carnet [27]. First, 200 µL of plant extract solution (at 20 mg mL^−1^ in water) was added to 200 µL water and 600 µL methanol. Then, 200 µL of the resulting solution was then added to 1 mL of 2% AlCl_3_, 6H_2_O methanolic solution, and 800 µL methanol. After vortexing for 2 min and incubating in darkness at RT for 15 min, the absorbance was measured at 430 nm (see Section 2.9 for the equipment). Standard calibration curve was realized with Q (0–35 mg L^−1^). The results were expressed as mg Q equivalent per gram of dry plant, and calculated as mean value ± 1 SD (*n* = 3). Herbal tea solution was replaced by water to set zero absorbance value.

### 2.13. Total Proanthocyanidin Oligomers Content (OPC)

HCl/n-butanol method was used to assess the total proanthocyanidin oligomers content (OPC) in plant extracts, as described by Porter et al. [28]. First, 200 µL of plant extract solution (at 20 mg mL^−1^ in water) was added to 200 µL water and 600 µL methanol. Then, 250 µL of the resulting solution was then added to 100 µL of 2% NH_4_Fe(SO_4_)_2_, 12H_2_O solution (in 2M HCl), and 3 mL of 95% n-butanol/HCl (95:5 *v*/*v*) solution. After vortexing for 2 min and incubating at 95 °C in an oil bath for 40 min, the resulting solution was cooled at RT. The absorbance was measured at 550 nm (see Section 2.9 for the equipment). Standard calibration curve was realized with CY (0–30 mg L^−1^). The results were expressed as mg CY equivalent per gram of dry plant and calculated as mean value ± 1 SD (*n* = 3). Herbal tea solution was replaced by water to set zero absorbance value.

### 2.14. UHPLC-DAD and UHPLC-ESI-MS Analysis

Plant extract solution (at 20 mg mL^−1^ in water) was prepared and then diluted five times with water before analysis by UHPLC-DAD (Thermo Scientific™ Dionex™ UltiMate™ 3000 BioRS equipment with WPS-3000TBRS auto sampler, TCC-3000RS column compartment set at 35 °C, and Chromeleon 7 software, from Thermofisher Scientific, Waltham, MA, USA) and by UHPLC-ESI-MS (Synapt G2-S equipment with ESI operating in resolution mode and MassLynx 4.1 software, from Waters Corp., Milford, MA, USA).

For both methods, a Kinetex C18 100A 100 × 2.1 mm, 2.6 µm column in association with a security guard ultra-cartridge was used (from Phenomenex Inc., Torrance, CA, USA). A binary solvent system was used. It consisted of water/formic acid (1‰, *v*/*v*) mixture as solvent A and acetonitrile/formic acid (1‰, *v*/*v*) mixture as solvent B. The gradient program started with 95% A, then A was progressively decreased to 0% in 30 min with a convex increase (curve 5 in Chromeleon 7). The flow rate of the mobile phase was set to 0.4 mL.min^−1^ and the injection volume was 20 µL.

For the analysis by UHPLC-DAD, the peaks were monitored at 280, 320, and 360 nm and the UV-Vis spectra of the various compounds were recorded between 200 and 500 nm.

For the analysis by UHPLC-ESI-MS, positive and negative ionization modes and fast DDA MS methods with automatic MS/MS intensity-based switching parameters, were used. The cone voltage, the extractor voltage, and the capillary voltage were set to 30 V, 3 V, and 2.4 kV, respectively. The source temperature and the desolvation temperature were 140 and 450 °C, respectively. Ions were scanned between *m*/*z* = 50 and *m*/*z* = 1200 to obtain MS spectra.

### 2.15. ESI(-) FT-ICR-MS Analysis

The dried extract was dissolved into 2 mL ultrapure water in glass vial and put for 2 min in an ultrasonic bath at RT. The resulting aqueous solution was recovered and transferred in a 2.5 mL vial for 2 min centrifugation at 14,000 rpm. The solution was then diluted 200 times in methanol for direct injection in the mass spectrometer (12T FT-ICR Solarix equipment and FTMS-Control V2.2.0 software, from Bruker Daltonics, Bremen, Germany). 

External calibration of the mass spectrometer was performed with arginine clusters (at 10 mg L^−1^ in methanol) prior to acquisition. The methanolic solution was infused at 2 µL min^−1^ flow rate in the ESI source (Apollo II, Bruker Daltonics, Bremen, Germany) that was used in negative-ion mode with a capillary voltage set at 3.6 kV. The flow rate, the drying gas temperature, and the nebulizer gas pressure were kept at 4 L min^−1^, 180 °C, and 2.2 bar, respectively. MS spectra resulting from 300 scans that were accumulated over a *m*/*z* 122–100 range and with a 4 megaword time-domain, were then processed using Data Analysis 5.0 software (Bruker Daltonics, Bremen, Germany). An internal calibration was realized using well-known C_x_H_y_O_z_ anions (composed of fatty acids and sugars), with mass accuracy values lower than 500 ppb. Lists of peaks were generated at signal-to-noise ratio ≥4 and then exported. Signals related to satellite and magnetron peaks were removed using an algorithm initially developed by Kanawati et al. [29]. As the samples were all analyzed in replicates, only *m*/*z* features observed at least 60% were kept and aligned in a matrix with 0.5 ppm tolerance. As previously reported [30], the achieved matrix was processed for assignment by computing the average *m*/*z* values with 0.5 ppm annotation tolerance. Eventually, CHO, CHON, CHOS, CHONS, and CHOCl molecular series were achieved. 

Perseus software was used to perform principal component analysis (PCA). The chemical composition description yielded for the different samples was detailed by means of heteroatom class distribution and van Krevelen diagrams representing the hydrogen-to-carbon and oxygen-to-carbon atomic ratios. It was possible to distinguish various biochemical compounds such as lipids, amino acids, carbohydrates, and polyphenols according to their location on the van Krevelen diagram [4,31]. Peaks were putatively assigned via MassTRIX with metabolite compounds observed in the *Arabidopsis thaliana* [32]. Deprotonated and chlorinated adducts were allowed for putative compound assignment within a 0.5 ppm window.

### 2.16. Enzymatic Activities Assays

#### 2.16.1. Hyaluronidase Capillary Electrophoresis Inhibition Assay

The effects of the different plant extracts were evaluated towards hyaluronidase activity using an optimized protocol based on a CE/PDA assay previously described by some of us [33]. Briefly, 25 µL of incubation buffer was preincubated with 10 µL of HA (at 4 mg mL^−1^) and 10 µL of plant extract (at 5 mg mL^−1^) at 37 °C for 10 min. Then, 5 µL of enzyme was added into the resulting mixture. The final concentration of the enzyme in the mixture was 0.2 mg mL^−1^. Reactions were incubated for 180 min at 37 °C, then they were stopped by increasing the temperature to 90 °C for 10 min using a water bath [33,34]. Results were compared to those obtained with a referenced inhibitor of hyaluronidase (EGCG) at 1 mg mL^−1^ in the mixture [34,35]. Control assays, where HA hydrolysis occurred normally in absence of plant extracts, were performed and stopped using the same protocol: 35 µL of incubation buffer solution was preincubated with an appropriate volume of HA (at 4 mg mL^−1^) at 37 °C for 10 min, then 5 µL of enzyme was added into the resulting mixture. All reactions were performed in triplicate.

A PA800+ CE apparatus equipped with a photodiode array detector and Beckman 32 Karat software (Sciex, Redwood City, CA, USA) was used for the analysis of the reaction mixtures and the aqueous raw extracts. Fused silica capillaries (from Polymicro Technologies, Phoenix, AZ, USA) were used with a total length of 57 cm (47 cm effective length) and 50 µm inner diameter. Detection wavelength was set to 200 nm. Between runs, the capillary was flushed with NaOH (for 5 min), water (for 0.5 min) and background electrolyte (BGE) (for 3 min) to ensure a good cleaning of the inner capillary surface. Rinse cycles were all carried out at 50 psi. Hydrodynamic injections were carried out from the anodic side of the capillary at 1.5 psi for 5 s. Separation voltage was performed in positive polarity mode at +15 kV. The corrected peak area (CPA), that was determined by the ratio of area to the migration time, was a reliable mean for the tetrasaccharide quantification (final product of HA hydrolysis). It was followed to assess the enzyme’s activity in the presence of plant extracts and compared to reactions carried out in absence of these extracts. The percentage of inhibition was calculated according to Equation (2):

(2) $$\%\;I=\left(1-\;\frac{A_{x}}{A_{0}}\right)\times 100$$

where *A_x_* and *A*_0_ are the CPA of tetrasaccharide formed in the presence and in the absence of plant extract, respectively.

Buffers and stock solutions for hyaluronidase CE assay: The incubation buffer (IB) was 2 mM sodium acetate (in deionized water) at pH 4.3 (adjusted with 1 M glacial acetic acid). The BGE was 50 mM ammonium acetate (in deionized water) at pH 8.9 (adjusted with 1 M ammonia). All buffers were filtered using hydrophilic PVDF syringe filters before use. HA, EGCG, BTH, and tetrasaccharide stock solutions were prepared at 10 mg mL^−1^ in IB and then diluted to the appropriate concentrations. Aliquots of 2 mg mL^−1^ were stored at −20 °C. Extract stock solutions were prepared at 5 mg mL^−1^ in deionized water, filtered using hydrophilic PVDF syringe filters, and stored at 4 °C. All buffer solutions were prepared daily and stored at 4 °C.

#### 2.16.2. Angiotensin-Converting Enzyme (ACE) Inhibition Assay 

The effects of the plant extracts on ACE activity were investigated using an ACE-kit WST. Plant extracts were prepared in deionized H_2_O at 13 mg mL^−1^ and then centrifuged at 2000 g for 10 min at RT. The supernatant was then recovered and stored at −20 °C before use. The concentration of the extracts was 1 mg mL^−1^ in the final reaction media.

The different reagents needed to run the ACE inhibition assay were prepared as instructed in the kit’s protocol. Briefly, the enzyme working solution was prepared by dissolving the contents of the vial labelled enzyme B with 2 mL deionized H_2_O and then adding 1.5 mL of enzyme B solution to the vial labelled enzyme A. The indicator solution was prepared by dissolving the contents of the vials labelled enzyme C and coenzyme in 3 mL deionized H_2_O each and then adding 2.8 mL of each to the vial labelled indicator solution. All solutions were stored at −20 °C. The ACE inhibition assay was carried out in 96-well microtiter plates. To screen the inhibitory effect of each extract, 20 µL of the sample solution, 20 µL of the 3-Hydroxybutylyl-Gly-Gly-Gly (3HH-GGG) substrate buffer solution, and 20 µL of the enzyme solution were added to the designated wells. The positive control consisted of substituting the 20 µL of the extracted sample solution with deionized H_2_O. The reagent blank consisted of substituting the 20 µL of the sample and the enzyme solutions with 40 µL of deionized H_2_O. Since the sample solutions were colored, sample blanks were carried out containing only 20 µL of the sample solution and 240 µL of deionized H_2_O. The plate was then incubated for 1 h at 37 °C in a thermostated oven (Heareus, Hanau, Germany) before adding 200 µL of the indicator solution to each well except those wells with sample blanks making the final volume of each well 260 µL. The plate was then incubated at RT for 10 min before reading mixtures’ absorbances at 450 nm using a Thermo scientific Multiskan GO UV/Vis microplate spectrophotometer (from Thermofisher scientific, MA, USA). ACE inhibition assays were performed in triplicates (*n* = 3) and plate readings in duplicates (*n* = 2). Percentage of inhibition of ACE by the plant extracts was calculated according to Equation (3):

(3) $$\%\;ACE\;inhibition=\frac{A_{blank1}\;-\;(A_{sample}\;-\;A_{blank3})}{\left(A_{blank1}\;-\;A_{blank2}\right)}\times 100$$

where *A_blank_*_1_ is the absorption of the positive control without any extracts, *A_blank_*_2_ is the absorption of the reagent blank without any extracts nor enzyme, *A_blank_*_3_ is the absorption of the sample blank, and *A_sample_* is the absorption of the reaction modulated by the plant extracts.

### 2.17. ABTS Antioxidant Assay

The antioxidant capacity of the various extracts was investigated according to the method described by Messaili. S. et al. [36]. Briefly, 7 mM of ABTS and 2.45 mM potassium persulfate were mixed and agitated in the dark for 16 h at RT to form the blue-green ABTS radical solution. The ABTS radical solution was then diluted with ethanol/water (25/75) at a 1:12.5 volume ratio. To carry out the antioxidant assays, 190 µL of the ABTS diluted solution was mixed with 10 µL of the extract or positive control solution in a 96 well-microtiter plate. Plant extracts were prepared in deionized H_2_O and their antioxidant capacities determined at 1, 0.1, and 0.01 mg mL^−1^. Similarly, Trolox was prepared at 1, 0.1, and 0.01 mg mL^−1^ in pure ethanol and used as a positive control. The absorbance of the plate was recorded at 734 nm after 30 min incubation in the dark at RT. The antioxidant assays were performed in triplicate (*n* = 3) and plate readings in duplicates (*n* = 2). The antioxidant activities of the various extracts and the positive control were assessed based on their ability to induce decolorization of the ABTS radical solution by electron transfer. This was manifested by a reduction of the absorbance compared to the absorbance of the ABTS radical solution. The percentage reduction in absorbance is calculated according to Equation (4):

(4) $$\%\;\mathrm{reduction}\;\mathrm{of}\;\mathrm{absorbance}\;=\left(1-\frac{A_{sample}}{A_{ABTS}}\right)\;\times 100$$

where *A_sample_* is the absorption of the mixture of the ABTS radical and extract samples or trolox and *A_ABTS_* is the absorption of the ABTS radical solution only.

Table 1 summarizes all the experiments realized on the three plants with the corresponding lot numbers, the extraction mode, and the granulometry.

## 3. Results and Discussion

The objective of the first part of this work was to investigate if the optimized extraction protocol that was previously developed for HAW, also stands for the extraction of BC and CA bioactive components. Therefore, different modes of extraction (infusion, ultrasonic, maceration, percolation, and microwave) were compared. For reasons of simplicity and to get a final optimized protocol that can be used by anyone, this study was intentionally restricted to water as extracting solvent. All the extraction protocols are described in the experimental part (see Section 2.3, Section 2.4, Section 2.5, Section 2.6, Section 2.7, Section 2.8). The kinetics of extraction were studied for both infusion and maceration extraction modes (see Section 2.9). The global mass extraction yields together with the contents in polyphenols (TPC), flavonoids (TFC), and proanthocyanidin oligomers (OPC) were determined for all extraction modes and for two plant granulometry (see Section 2.11, Section 2.12, Section 2.13). All extractions were carried out in triplicate (three independent extractions) to ensure the reproducibility of the measurements. UHPLC-ESI-MS, ESI(-)-FT-ICR-MS, and enzymatic activity analyses were also performed to get a better insight in the differences of chemical compositions and pharmacological properties between the three plants.

### 3.1. Influence of the Extraction Mode and of the Plant Grinding on the Global Extraction Yields and on the Kinetics of Extraction 

The kinetics of extraction were investigated for infusion and maceration of CA and BC, both on raw and ground plants, by monitoring the UV absorbance for 30 min at 198 nm (Figure 1). This simple analytical method provides interesting information about the kinetics of extraction of the water-soluble components from the plants. Low UV wavelength is used to detect the largest number of extracted chemical compounds. In parallel to the UV monitoring, but on independent experiments, extraction yields (in mass % of solid extract compared to the dry plant initial mass) were determined by evaporation and lyophilizing of the whole extract at 10 min (or 30 min) extraction times (Table 2).

For raw materials, the kinetics of extraction is much faster for infusion mode (see Figure 1A, open symbols) compared to maceration at 60 °C (see Figure 1B), as already observed for HAW. This can be quantitatively assessed by the decrease of the time *t*_70%_ to get 70% of the absorbance value at highest extraction time at 30 min (Table 2): from 7 min (resp. 15.5 min) for maceration at 60 °C of CA (resp. BC) to 2 min (resp. 8.5 min) for infusion of CA (resp. BC). As for the extraction yield at 10 min (Table 2), the increasing order of extraction yield for raw CA and BC was as follows: percolation < maceration at 60 °C < infusion < US < MW. The same order was found for raw HAW extraction, except for the percolation mode that was the most efficient in that latter case. For the three plants, infusion was always more performant compared to maceration at 60 °C, and this was especially true at 10 min extraction for CA (8.4% extraction yield for maceration vs. 15.2% for infusion).

Similar extractions were carried out on ground (with 1 mm mesh size grinder) BC and CA, using the same lots as the previous experiments. As already shown before for ground HAW [4], ground BC and CA led to much faster kinetics of extraction (see Figure 1A for infusion and Figure 1B for maceration, plain symbols) with *t*_70%_ lower than 1.5 min for both extraction modes (see Table 2). Extraction yields were very similar at 10 and 30 min extraction times (30–33% for CA and BC, and 20–25% for HAW [4] for all extraction modes), due to fast extractions. These yields were much higher compared to those obtained with raw materials (+40% for CA and HAW infusion and +100% for BC infusion, at 10 min extraction time, see Table 2 and [4]). This gain on the extraction yield is especially remarkable for BC, most likely because of the large size of the raw leaves for which the grinding ensures a considerable increase of the specific surface compared to raw material. The impact of grinding on the extraction yields tends to decrease with the extraction time but still remains substantial at 30 min (+28% for CA infusion, +60% for BC infusion and +40% for HAW infusion [4]).

As a conclusion of this part, for all the plants considered in this work and in [4] (i.e., CA, BC and HAW), the extraction by infusion was found to be an efficient and the easiest way to extract the water-soluble components, in less than 2 min provided that the plant was ground. It is worth noting that between the lowest extraction yield at 10 min (percolation on raw BC, 7.4%) and the highest value (MW on ground BC, 33.1%), a factor of ≈4.5 was found on the extraction yield, demonstrating the great impact of the extraction protocol. 

### 3.2. Quantification of Total Flavonoid, Polyphenol, and Proanthocyanidin Contents

After their extraction in water, all the plant extracts were evaporated, freeze-dried, and kept at −18 °C in the dark. Colorimetric methods were used to determine the total amounts of flavonoid (TFC), polyphenol (TPC), and proanthocyanidin oligomers (OPC) contents (see Section 2.11, Section 2.12, Section 2.13) that were expressed as equivalent content in Quercetin (Q) for TFC, Gallic acid (GA) for TPC, and Cyanidin (CY) for OPC. Table 2 gathers the numerical values for both 10 and 30 min extraction times (*n* = 3 repetitions on three independent extractions).

As expected, and as observed for the global extraction yield, the extraction yields of TFC, TPC, and OPC were much higher for ground material than for raw material. The gain in extraction due to the grinding is even more important for the phenolic components than for the global yield: TFC (+60% CA, +180% BC and +50% HAW), TPC (+50% CA, +200% BC and +70% HAW), and OPC (+80% CA, +330% BC and +210% HAW).

Regarding the different extraction modes, the MW extraction mode generally gives the highest extraction yields, in good agreement with the literature [4,22,23,24,25,37,38,39,40]. On raw plants, the extraction mode can have a great impact, with an enhanced extraction factor (EF) at 10 min extraction of about 1.7 for CA (resp. 2.8 for BC) between the worst (percolation) and the best (MW) modes. However, the differences between the extraction modes are very limited for ground materials (5–10% differences on TFC, TPC, and OPC extraction yields, see Table 2). Therefore, and as already observed for HAW [4], the grinding of the dry plant is the most important factor to increase the extraction yields, and the infusion mode is the simplest and most accessible extraction mode to be selected.

The dry extract amount and the TFC, TPC, and OPC contents issued from a single 10 min infusion of 2.5 g of 1 mm ground plant in 250 mL water at 500 rpm were determined. One infusion brings about 795 mg/760 mg/555 mg of dry extract for CA, BC, and HAW respectively. These extracts contain 43 mg/118 mg/82 mg equivalent GA (TPC), 11.1 mg/11.9 mg/8.6 mg equivalent Q (TFC), and 1.6 mg/5.9 mg/9.8 mg equivalent CY (OPC) (Table 2 and [4]). It is worth noting that BC extracts contain much more phenolic compounds than the two other plants, while HAW extracts contain much more proanthocyanidin oligomers than the two other plants. CA and BC extracts contain similar amounts of flavonoids.

### 3.3. Characterization of the Plant Granulometry and Optimized Easy-To-Use Infusion Protocol

**Figure 2:**
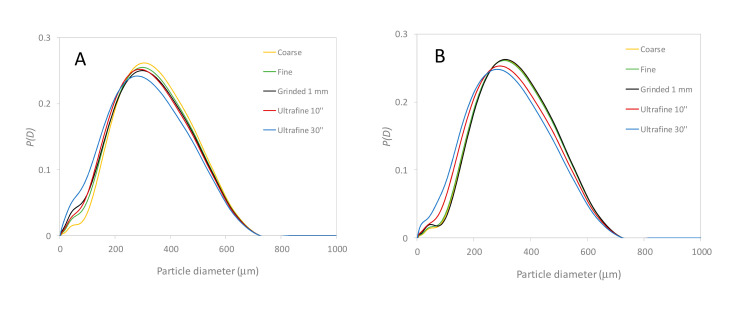

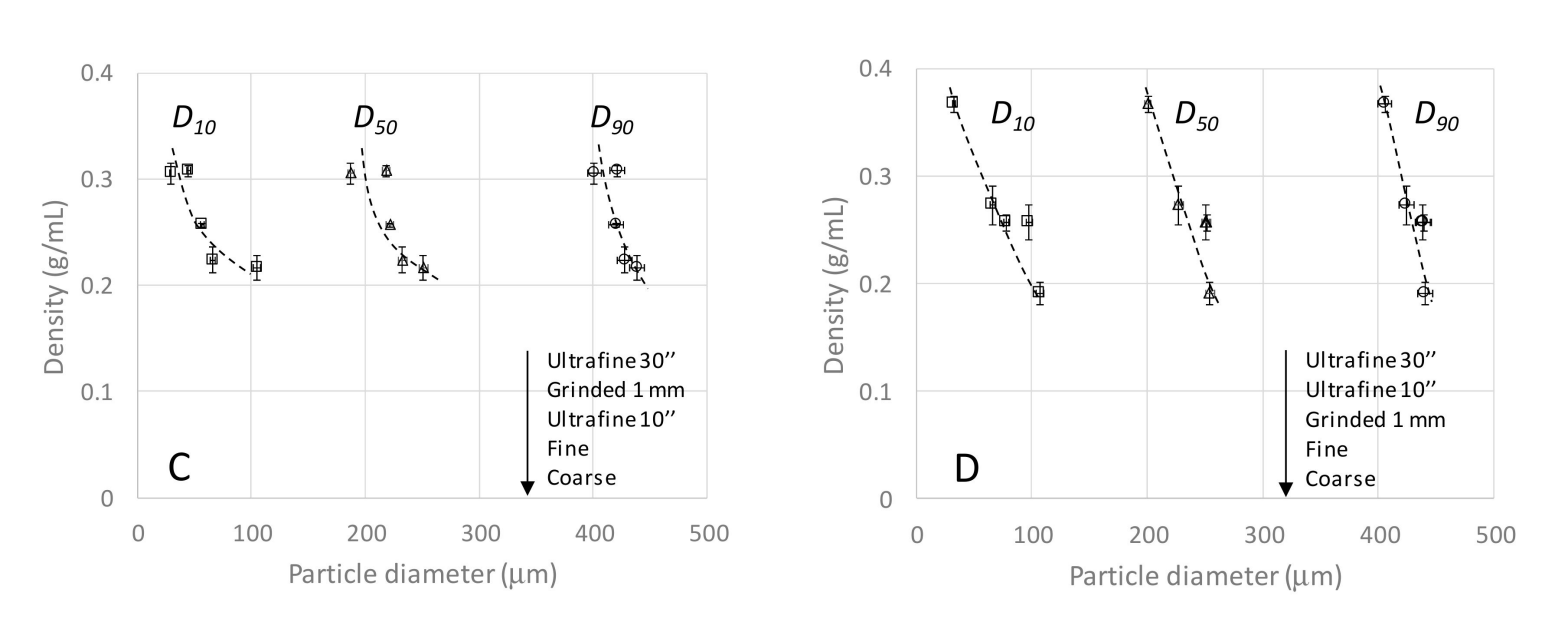
Relative size distributions of the raw and ground CA (**A**) and BC (**B**) plant materials obtained by laser granulometry in dry mode and variation of the density of CA (**C**) and BC (**D**) plant materials as a function of the particle diameter. *D*_10_ (□), *D*_50_ (∆), and *D*_90_ (◯) with *D_x_* being the tenth decile of the distribution. See Section 2.2 for more details on the experimental conditions. Lot number for CA: 559980. Lot number for BC: 55870.

An easy way to grind the plants in affordable and reproducible manner, was used. Two electric coffee grinders were employed: a KG79 Delonghi grinder (using ‘coarse’ and ‘fine’ positions) and a MK6003 Bosch grinder (using 10 and 30 s grinding times by manually shaking the grinder simultaneously). A MF10 basic Ika laboratory grinder was used with 1 mm grid size too. Figure S1 shows the pictures of the CA and BC materials on a millimeter paper, before (raw) and after the previously described different grinding protocols. The plant density of the ground material is also given for each picture and was simply measured with a graduated test tube (see Section 2.2). To get better quantitative data, laser granulometry in dry phase was used to determine the size distribution of the plant particle (see Section 2.2 for the experimental part, and Figure 2A,B for the size distributions of CA and BC, respectively). Broad volume size distributions (data given in diameter) were obtained with typically a bimodal curve having sizes ranging between 10 and 80 µm for the first mode (or as a peak shoulder), with a main mode between 200 and 250 µm, and maximum size up to 500 and 700 µm. The correlation between the ground plant density and the average particle diameters are presented in Figure 2C,D for different deciles of the distribution (*D*_10_ is the first decile, *D*_50_ is the median value, and *D*_90_ is the ninth decile). From these correlations, we can roughly estimate the minimum density required to get an adequate granulometry to optimize the plant extraction. This could be very useful in practice (for instance at home) when granulometry measurement is not available. To get a granulometry corresponding to the ‘fine’ position of the Delonghi (Model KG79) grinder, or even lower granulometry, a plant density equal or higher than 0.22 g mL^−1^ for CA, 0.26 g mL^−1^ for BC, and 0.27 g mL^−1^ for HAW is required. As a first approximation, the value of 0.25 g mL^−1^ can be retained for the three plants. 

Since the infusion mode was found to be the easiest way to perform the water-based extraction on ground materials, the optimized protocol already used for HAW [4] was also implemented for the extraction of CA and BC. This easy-to-use-at-home protocol is based on the following steps: (1) grinding 2.5 g raw dry plant using a commercially available basic grinder just before performing the infusion (‘fine’ granulometry, density of ground plant about 0.25 g mL^−1^ or higher); (2) pouring 250 mL boiling water onto the ground plant in a French-press coffee maker without any infusion bag, the piston allows to push the residual solid parts of the plant to the recipient bottom; (3) vigorous manual stirring of the mixture; (4) waiting at least 3 min; (5) pressing the French-press filter to retain the remaining solid part of the plant before serving. Using this protocol, the global extraction yields are optimized and repeatable (25.9% for CA; 28.6% for BC and 21–22% for HAW, see Table 3). The same stands for the quantities of extracted TFC, TPC, and OPC (Table 3). The impact of the plant granulometry on the extraction yields was not investigated in this work for CA and BC; however, this study was previously performed for HAW showing that ‘fine’ position granulometry was sufficient to get optimal extraction yields [4].

In the laboratory conditions of infusion (Section 2.3), temperature decreased in the reactor after 30 min extraction at 500 rpm from about 90 to 40 °C and for a volume of water of 250 mL. The drinkable temperature (60 °C) to elude any side effect on the health [41,42] was reached after 10 min extraction. In practice, we find it interesting to study the temperature profile that we would encounter at home by infusion in the French press Bodum^®^ (see Section 2.8), for two volumes of water (250 mL corresponding to a mug; or 405 mL for a bowl, see Figure S7). Cooling down the infusion at drinkable temperature (60 °C) requires much longer times (8 min for 250 mL, and 12 min for 405 mL) than the time required for the extraction (about 3 min). To speed-up the cooling process after the 3 min extraction, and drink safely without waiting too long, it is possible to add ice cube(s) in the French press Bodum^®^, as shown in Figure S7. For 250 mL of water, only one ice cube (≈23.4 g) is necessary to reach 60 °C; with a full melting of the ice cube within 1 min, leading to a total preparation time of about 4 min (at home). For 405 mL of water, two ice cubes (≈23.4 g each) are required to reach 60 °C; with a total preparation time of about 5 min.

### 3.4. Chemical Composition of the CA and BC Plant Extracts Investigated by UHPLC and Its Coupling with MS

**Figure 3:**
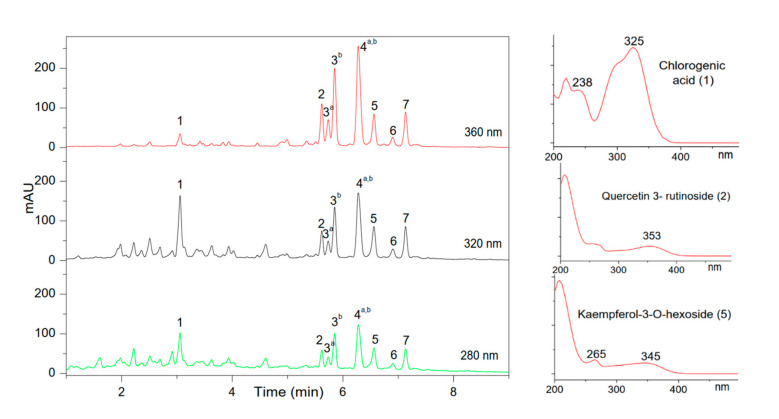
UHPLC profiles of BC extracts issued from infusion Bodum^®^ extraction mode for ‘fine’ ground BC material monitored at 280, 320, and 360 nm. Typical UV spectra of the main compounds are given on the right. UV spectrum of Chlorogenic acid represents hydrocinnamic acid derivatives, UV spectrum of Quercetin 3- rutinoside (2) represents Quervetin 3-O-galactoside (3^a^), Quercetin 3-O-glucoside (3^b^), and Quercetin-3-6-malonyl-glucoside (4^a^), UV spectrum of Kaempferol-3-O-hexoside (5) represents Kaempferol-3-O-rutinoside (4^b^), Kaempferol-malonylglucoside (6), and Kaempferol-malonylglucoside isomer (7). See Table 4 for peak ID. Lot number: 55870. See Figure S4 for UHPLC profiles of other lots.

**Figure 4:**
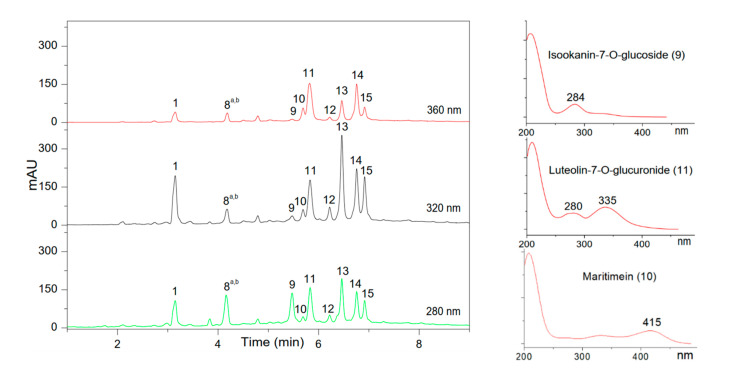
UHPLC profiles of CA extracts issued from infusion Bodum^®^ extraction mode for ‘fine’ ground CA material monitored at 273 nm. Typical UV spectra of main compounds are given on the right. UV spectrum of Chlorogenic acid (1) represents hydrocinnamic acid derivatives (12, 13, 15), UV spectrum of Isookanin-7-O-glucoside (9) represents Eriodicyol-7-O-glucoside (8^a^), UV spectrum of Luteolin-7-O-glucuronide (11) represents 6,8-C,C-diglucosyl-apigenin (8^b^) and Apigenin-7-glucuronide (14). See Table 5 for peak ID. Lot number: 559980. See Figure S5 for UHPLC profiles of other lots.

To get a better insight into the differences between CA and BC (as well as with previously published HAW results), positive mode UHPLC-ESI-MS and reversed phase UHPLC analysis were carried out (see Section 2.14) as described in [43]. A detailed list of the samples studied in this work by UHPLC-ESI-MS is given in Table 1. The UV chromatographic profiles obtained for the infusion extracts (issued from the infusion of ‘fine’ ground materials using the optimized easy-to-use infusion protocol, see Section 2.8) of BC and CA, are presented in Figure 3 and Figure 4 respectively, at three different wavelengths, 280 nm for flavan-3-ols and flavanones, 320 nm for hydroxycinnamic acids, and 360 nm for flavonols (see also Figure S3 for the chemical structure of all identified compounds and Figures S4 and S5 for the profiles of the other lots). Table 4 and Table 5 contain the list of the identified components of BC and CA with names and molar masses, respectively.

Among nine major peaks detected in BC (see Figure 3), two of them (peak 1 = Chlorogenic acid, and peak 3^b^ = Quercetin 3-O-glucoside (Isoquercetin)) were unambiguously identified by comparing the UV spectrum, the retention times, and MS or MS/MS data with the reference standards used in a previous study [4]. The peaks 2, 3^a^, 4^a^, 4^b^, 5, 6, and 7 were tentatively assigned, respectively to: Quercetin 3-rutinoside (2), Quercetin 3-O-galactoside (3^a^), Quercetin-3-6-malonyl-glucoside (4^a^), Kaempferol-3-O-rutinoside (4^b^), Kaempferol-3-O-hexoside (5), Kaempferol-malonylglucoside (6), Kaempferol-malonylglucoside isomer (7); based on the data from literature review [9,29,30,31,44,45,46], UV spectra maximum values and their fragmentation patterns from ESI-MS(+/−) and MS/MS data, as presented in Table 4.

Chemical composition of CA extracts has been much less investigated in the literature than for BC or HAW. Among 10 major peaks detected in CA (see Figure 4 and Table 5), one of them (peak 1, Chlorogenic acid) was unambiguously identified by comparing the UV spectra maximum values, the retention times, and ESI-MS(-), MS/MS data with the reference standards used in our previous study [4]. The peaks 8^a^, 8^b^, 9, 10, 11, 12, 13, 14, and 15 were tentatively identified, respectively to: Eriodicyol-7-O-glucoside (8^a^), 6,8-C,C-Diglucosylapigenin (8^b^), Isookanin-7-O-glucoside (Flavanomarein) (9), Maritimetin-6-O-glucoside (Maritimein) (10), Luteolin-7-O-glucuronide (11), Di-caffeoylquinic acid (12), Apigenin-7-glucuronide (14), Di-caffeoylquinic acid isomers (13, 15); based on the data from the literature [17,32,33,34,35,36] and their fragmentation patterns from ESI-MS(-), MS/MS data as presented in Table 5. Compound peaks 8^a^, 9, and 10 have been described by Shimokoriyama and Honore-Thorez [18,47] and those peaks showed UV maximum absorption bands at 282, 284, and 414 nm, respectively, very similar to Eriodicyol-7-O-glucoside, Flavanomarein, and Martitimein, respectively. The MS/MS spectrum of peaks 8^a^, 9 revealed three fragments corresponding to Eriodicyol-7-O-glucoside (*m*/*z*, 287.0532, 151.0001, 135.048) and Flavanomarein (*m*/*z*, 287.0532, 269.0437, 135.0055, 135.0429). Thus, peaks 8^a^, 9 were tentatively identified as Eriodicyol-7-O-glucoside, Flavanomarein. Additionally, the MS/MS spectrum of *m*/*z* 447 ([M−H]^−^) presents the fragments corresponding to *m*/*z* 285 (Maritimetin) and another *m*/*z* 151, *m*/*z* 135 and the molecule ion at m/z 447 ([M−H]^−^) in agreement with Maritimein glucoside. Thus, peak 10 was tentatively identified as Maritimein. 6,8-C,C-diglucosylapigenin, Luteolin-7-O-glucuronide, Apigenin-7-glucuronide were described for the first time in CA. Based on the literature on *Chrysanthemum* species [48,49,50], peak 8^b^ presented the UV maximum absorption band at 267 nm, the molecule ion at *m*/*z* 593 ([M−H]^−^) and other fragment ion at *m*/*z* 473, 353, 191; peak 11 revealed the UV maximum absorption band at 280 nm, 335 nm, molecule ion at *m*/*z* 461 ([M−H]^−^); peak 14 showed the UV maximum absorption band at 266 nm, 334 nm, the molecular ion at *m*/*z* 445 ([M−H]^−^) in agreement with 6,8-C,C-Diglucosylapigenin, Luteolin-7-O-glucuronide, and Apigenin-7-glucuronide, respectively. The MS spectra of peaks 12, 13, and 15 showed the same molecular ion at *m*/*z* 515 ([M−H]^−^), and another fragment ion at *m*/*z* 191, 179, 173, 135. Based on the literature [49,50,51], those were described as a characteristic of a Dicaffeoylquinic acid. As there are six isomers of Dicaffeoylquinic acid in nature, those were thus tentatively identified as Dicaffeoylquinic acid isomers.

### 3.5. Global Composition and Differences in Chemical Composition between Plants Achieved by ESI FT-ICR-MS in Negative Mode 

In this section, the discussion is essentially focused on the ‘fine’ ground samples obtained with the infusion mode using the optimized protocol (see Section 2.8). A detailed list of the samples studied by ESI FT-ICR-MS is given in Table 1. MS spectra obtained for these samples are given in Figure S2 in duplicate (on two independent extractions), with a description of the global composition in heteroatom classes and van Krevelen diagram. The latter is obtained by plotting the achieved raw formulae as a function of their O/C and H/C ratios. It helps to distinguish between different biochemical families such as amino acids, lipids, polyphenols, and carbohydrates depending on the plot location [31,52].

Heteroatom class distributions and van Krevelen diagrams obtained for BC and CA show similar fingerprints (Figure S2), as observed in the previous study with HAW samples [4]. CHO molecular series is predominant irrespective of the sample type. Its representation on the van Krevelen indicates some carbohydrates, polyphenols, hydrolysable tannins, and lipids. Concerning the CHON species, they correspond to amino acids, small peptides, and likely amino sugars. All the ESI(-) FT-ICR mass spectra present intense peaks at *m*/*z* 191.0561 and *m*/*z* 353.0878 that could, respectively, correspond to the deprotonated form of Quinic acid and Chlorogenic acid or 5-O-Caffeoylquinic acid.

**Figure 5:**
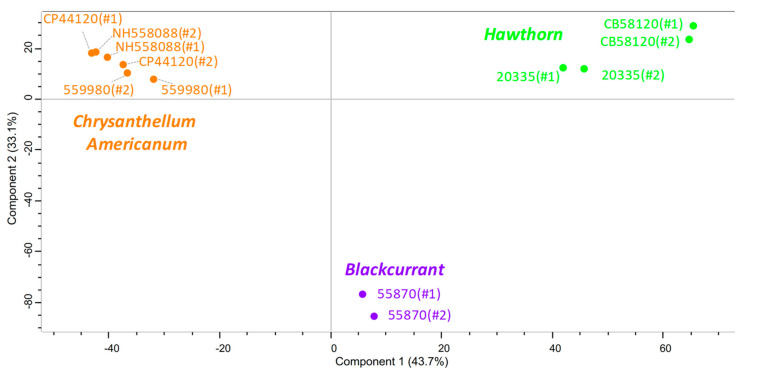
PCA score plot of all mass features measured by ESI(-) FT-ICR MS issued from HAW (green, lot numbers: CB58120 and APC27031904), BC (purple, lot number: 55870), and CA (orange, lot numbers: 559980, CP44120 and NH558088) extracts. The annotations #1 and #2 refer to the replicate number.

**Figure 6:**
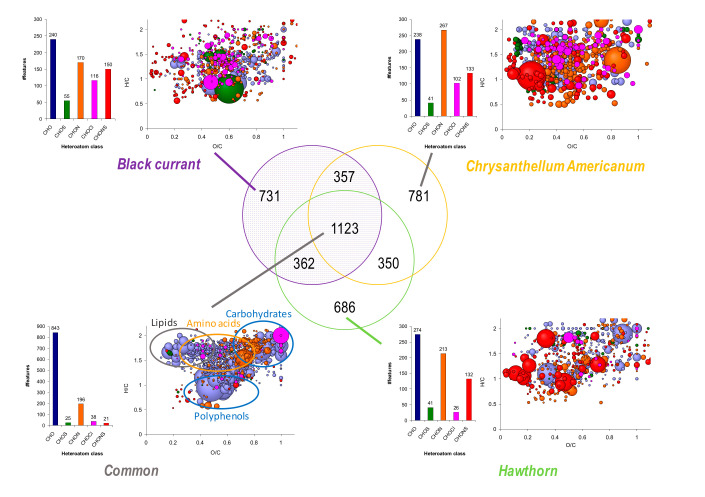
Venn diagram achieved from BC, CA, and HAW samples analyzed in ESI(-) FT-ICR MS. Heteroatom class distribution and van Krevelen diagram concern features specifically and commonly observed in all samples. Lot numbers: see Figure 5.

Nevertheless, as illustrated by the principal component analysis (PCA) score plot, there are some compositional differences between the samples depending on the plant (Figure 5). Therefore, a Venn diagram was performed to highlight differences in the chemical composition of the samples depending on the plant type (Figure 6). For each plant, about 2500 hints were obtained, among which about 1100 hints (25% of all the features) are common to all three plants; about 350 hints are common to any group of two plants; about 700 hints (about 15% of all the features) are specific to each plant. 

The van Krevelen diagram of the features specific to BC samples evidences CHO species relative to carbohydrates, polyphenols, terpenoids, and fatty acid-like species. CHOCl components cover the same areas. CHON and CHONS components are also well represented with some carbohydrates, polyphenols, and possibly some amino sugars. The most intensively detected species in these samples are mainly flavonoids, diterpenes, and, to a lesser extent, fatty acids and carbohydrates (Table S1). The features specifically observed in CA are mainly from CHON and CHO classes. Concerning the CHO species, the corresponding plots on the van Krevelen diagram suggest some polyphenols or flavonoid species (such as e.g., maritinein derivatives). Moreover, some fatty acid-like species, which likely possess some hydroxyl or carbonyl functions due to their higher O/C values, and terpenoid components are also observed. The most represented putative features are relative to terpenes, flavonoids, and other minor classes (see Table S1). For the HAW samples, several specific CHO species are highlighted, which are relative to carbohydrates and polyphenols according to the corresponding van Krevelen diagram. Within the CHON class, a few amino sugars are observed. Among the yielded putative compounds, of highest intensity, there are mainly some amino acids or derivatives, and some flavonoids (Table S1). Eventually, the van Krevelen diagram achieved for all common species shows a chemical fingerprint, very close to those achieved in the different samples (Figure S2) and in our previous work [4]. This chemical composition is dominated by CHO series, followed by the CHON one. The CHO compounds refer to lipids, carbohydrates, hydrosoluble tannins, and polyphenols. Regarding the CHON class, it involves amino acids, small peptides, and amino sugar-like species. The putative species attributed via MassTRIX are gathered in Table S1.

Additionally, the contributions specific to each plant sample were compared to formula of compounds identified in HAW for putative assignments [43,53,54]. Thus, no hints were obtained for BC sample whereas it seems that Oleanolic or Ursolic acid is specifically observed in CA samples (Table S1). For the HAW samples, two putative amino acids and three organic acids matched. However, 38 hints were found in the common features, which correspond to carbohydrates and organic acids but mostly to flavonoid-based components. Apigenin-7-glucuronide was putatively found in both HAW and CA samples whereas formula of Quercetin-3-6-malonyl-glucoside was observed in BC and HAW samples.

This approach ensured highlighting specific compounds in regard to their plant origin. Even if 25% of the features are commonly observed in all the samples, some differences can be evidenced. The focus was put on these compositional variations by means of graphical representations and putative assignments of the corresponding features.

### 3.6. Enzymatic Activities

The effects of the extracts obtained from three different plants on hyaluronidase and Angiotensin-converting enzyme (ACE) activities were assessed. For all assays, the extract concentration in the final reaction medium was 1 mg mL^−1^. All plant extracts were obtained by the optimized easy-to-use infusion protocol (see Section 2.8). Briefly, 2.5 g of dry plant was infused for 3 min in 250 mL boiling water before lyophilization. The obtained dry extracts were denoted as follows: (i) HAW extracts (Lot n° 20335, ground 1 mm, #1/#2 and Lot n° CB58120, ground ‘fine’, #1/#2), (ii) BC extracts (Lot n° 55870, ground ‘fine’, #1/#2) and (iii) CA extracts (Lot n° 559980, ground ‘fine’, #1/#2).

#### 3.6.1. Hyaluronidase CE Inhibition Assay

**Figure 7:**
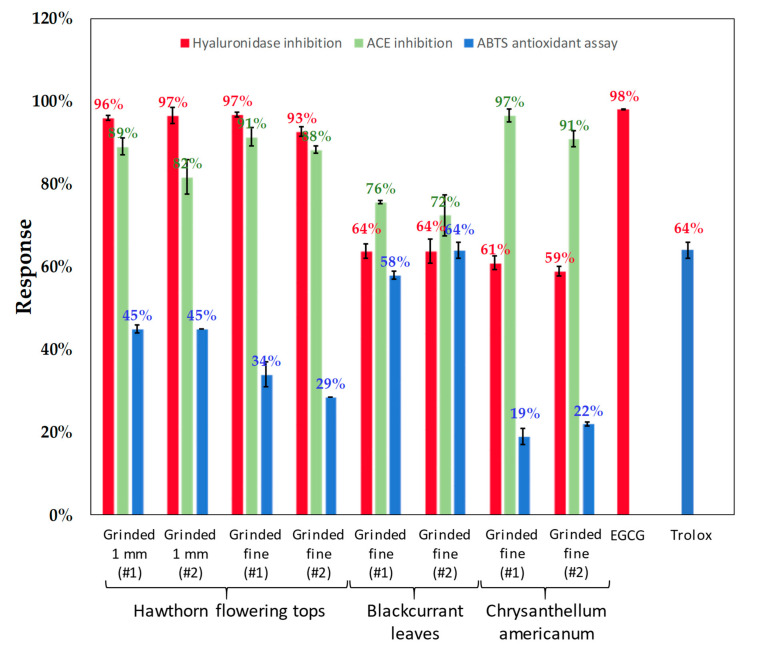
Percentage of inhibition of tested enzymes: hyaluronidase (in red) and ACE (in green) and ABTS antioxidant assay (in blue) in the presence of different extracts obtained from HAW, BC, and CA. For the enzyme inhibition assays, the plant extracts were screened at 1 mg mL^−1^ and the inhibition percentages of hyaluronidase and ACE were calculated according to Equations (2) and (3), respectively. The antioxidant capacities of the plant extracts were determined at 0.01 mg mL^−1^ and calculated according to Equation (4). The absorbance of the multiwell plates was read twice for both ACE inhibition and ABTS antioxidant capacity assays and the average of obtained results were plotted. All assays were carried out in triplicates (*n* = 3). Plant extracts were obtained from HAW (#1/#2, ground 1 mm, lot n°20335 and #1/#2, ground ‘fine’, lot n°CB58120), BC (#1/#2, ground ‘fine’, lot n°55870) and CA (#1/#2, ground ‘fine’, lot n°559980). EGCG, hyaluronidase referenced inhibitor, showed 98% hyaluronidase inhibition at 1 mg mL^−1^, and Trolox, an antioxidant reference, demonstrated 64% antioxidant capacity at 0.01 mg mL^−1^. Both references were used to validate the methods (more details in Table S2).

Hyaluronidase is well known for its involvement in numerous physiological and pathological processes such as skin aging, cancer progression, and inflammation by catalyzing the hydrolysis of hyaluronic acid (HA), a major compound of extracellular matrix [55,56]. The modulation of hyaluronidase activity in the presence of different plant extracts at a final concentration of 1 mg mL^−1^ was investigated by following the formation of tetrasaccharide and the percentage of inhibition was calculated according to Equation (2) (see Section 2.16). Results obtained with different extracts are reported in Figure 7 and Figure S6, where, for each extract, the average inhibition percentage of three enzymatic reactions (*n* = 3) was calculated as well as their standard deviation. The absence of any interferents with the tetrasaccharide peak was also verified by injecting the raw aqueous extracts at 1 mg mL^−1^ using the same capillary electrophoresis (CE) method described in Section 2.16.1.

Results reported in Figure 7 showed an overall reduction in hyaluronidase activity by all of the tested extracts obtained from the three different plants. An interesting inhibition, superior to 90%, was reported for HAW extracts similar to the inhibition observed with the referenced hyaluronidase‘s inhibitor, epigallocatechine gallate (EGCG), at the same concentration in the reaction media. The inhibition by BC and CA extracts was lower; 64% and 60%, respectively. These differences in inhibition effect towards hyaluronidase are mainly correlated to the subclasses of polyphenols and flavonoids identified in each extract [57,58,59] and not to the sum of TPC and TFC extracted mass. This is clearly observed with BC leaves which demonstrated the lowest hyaluronidase inhibition despite having the highest TPC and TFC amongst all extracts. In fact, BC extracts are rich in Quercetin and Kaempferol isomers (as reported in Table 4) both of which are flavonoids known to have moderate to low inhibitory effect towards hyaluronidase. Indeed, Gonzalez-Pena et al. [58] reported only 27% hyaluronidase inhibition by Quercetin at 0.23 mg mL^−1^ (750 µM) and Mohamed et al. [57] showed only 8% hyaluronidase inhibition by Kaempferol at 1 mg mL^−1^.

#### 3.6.2. ACE Inhibition Assay

Angiotensin-converting enzyme (ACE) catalyzes the cleavage of angiotensin I to angiotensin II resulting in elevation of blood pressure through vasoconstriction [60,61]. ACE inhibitors block the production of angiotensin II and thus help in the regulation of blood pressure [62]. Certain naturally occurring molecules such as flavonoid and non-flavonoid polyphenols are abundant in plants and have been described for their ACE inhibitory potential [63].

The results reported in Figure 7 demonstrated an interesting ACE inhibitory trend for all of the tested plant extracts. The percentage of ACE inhibition ranged roughly between 72% and 97%. HAW extracts demonstrated strong ACE inhibition (81–92%). HAW extracts have been described as an effective treatment of mild hypertension [64] and moderate heart failure [65,66,67,68,69,70,71]. The extracts of leaves and flowers (flowering tops) were shown to induce cardiovascular effects such as vasodilation and endothelial protection [64,72]. The plant lot did not appear to influence ACE inhibition as both extracts of flowering tops demonstrated a similar range of inhibition. BC extracts demonstrated the lowest percentage of ACE inhibition (72–76%) compared to HAW and CA extracts. Indeed, blackcurrant leaves have been shown to induce blood vessel dilation through the activation of endothelial nitric oxide synthase and not through the inhibition of ACE [73]. CA extracts demonstrated the highest ACE inhibition (91–97%). To our knowledge, CA extracts have not been previously described as ACE inhibitors. *Chrysanthellum* plants, however, have been reported to have a hypotensive effect through their intravenous use [25,74]. For the sake of comparison, it is worthy to note that captopril, a sulfhydryl-containing analog of proline prescribed as treatment of hypertension, was found to completely inhibit ACE at a low concentration close to 0.017 mg mL^−1^ [75], 58-fold lower than the one tested in this work, 1 mg mL^−1^.

The baseline inhibition of ACE by all three plant extracts may be linked to the presence of certain compounds in all of these extracts. For example, Chlorogenic acid demonstrated 88% ACE inhibition at 0.167 mg mL^−1^ (IC_50_ = 310.5 µM) [76] and Galloylglucoses 1,2,3,6-Tetra-O-galloyl-β-D-glucose and 1,2,3,4,6-Penta-O-galloyl-B-D-glucose demonstrated 42% ACE inhibition at 0.1 mg mL^−1^ (IC_50_ = 101 µM) and 49% ACE inhibition at 0.075 mg mL^−1^ (IC_50_ = 73 µM), respectively [77]. The differential inhibition between CA (≈95%) and BC (≈75%) may be explained by the specific phytochemical contents determined by ESI(-)FT-ICR-MS of each individual extract. Comparing the heteroatom class distribution and van Krevelen diagrams of all three extracts (Figure 6) shows that blackcurrant leaves had the lowest number and levels of CHON features (170) compared to CA which had the highest number of these features (267) as well as the largest bubble sizes. The sizes of the bubbles in the van Krevelen diagrams represent peak intensities of species detected by ESI(-) FT-ICR. The levels and numbers of amino acids, small peptides, amino sugar like-species (CHON) in CA was the highest compared to the other two extracts. This may suggest that the ACE inhibition potential of the extracts is related to their quantity of CHON species.

### 3.7. ABTS Antioxidant Assay

Antioxidant activities of both HAW [78] and BC leaves [79] have been previously reported. To our knowledge, this is the first report of CA antioxidant capacity. The extracts’ antioxidant capacities were assessed based on their ABTS radical scavenging abilities (see Section 2.17) and Trolox a known antioxidant compound was used as positive control and results were compared to its antioxidant capacity. As for hyaluronidase and ACE assays, the antioxidant capacities of extracts and Trolox were investigated first at 1 mg mL^−1^ for comparison. At this concentration, all of the tested extracts demonstrated high antioxidant capacities equal to that of Trolox (≈100%). As a result, new assays were conducted at two lower concentrations 0.1 and 0.01 mg mL^−1^. At 0.1 mg mL^−1^, HAW extracts (lot n° 20335, #1/#2 and lot n° CB58120, #1/#2) and BC extracts (lot n° 55870, #1/#2, ground ‘fine’) showed high antioxidant capacities equal to that of Trolox once again (95%), while CA extracts (lot n° 559980, #1/#2) had only 64% of antioxidant capacity. 

At 0.01 mg mL^−1^, the obtained results reported in Figure 7 show that Trolox had an antioxidant capacity equal to 64% at this low concentration. BC extracts demonstrated the highest antioxidant capacities (58% and 64%, respectively) compared to the other extracts. More interestingly, the antioxidant activity of BC turned out to be equal to the antioxidant capacity of the referenced compound Trolox. On the other hand, the lowest antioxidant capacity was observed again with the extracts of CA (19% and 22%, respectively). Finally, HAW extracts demonstrated a range of antioxidant capacities between 28% and 45%. This difference in antioxidant capacities of the three plant extracts can be correlated to the TPC and TFC extracted contents. In fact, BC extracts had the highest antioxidant capacity and highest contents of TPC and TFC while CA extracts presented the lowest contents of TPC and TFC and thus the lowest antioxidant capacity.

## 4. Conclusions

This work demonstrated that for the three plants considered (i.e., CA, BC, and HAW), the easy-to-use-at-home protocol of extraction by infusion was an efficient and the easiest way to extract the water-soluble components, in less than 3 min provided that the plant was ground. This protocol is based on the grinding of 2.5 g of raw dry plant, the infusion for 3 min with 250 mL of boiling water in a French-press coffee maker. Using this protocol, the global extraction yields are optimized and repeatable (25.9% for CA; 28.6% for BC and 21–22% for HAW, expressed as mass proportion of the extracted compounds relative to the dry plant). A10 min cooling is required before consumption to reach drinkable temperature (60 °C); but the process can be easily speed-up, down to 4 min by adding one small ice cube to the preparation. 

About the comparison of the chemical composition between the three plants, the main result was obtained by FT-ICR-MS. About 2500 hints were obtained for each plant, among which about 1100 hints (25% of all the features) are common to all three plants. About 350 hints are common to any group of two plants, and about 700 hints (about 15% of all the features) are specific to each plant. It is worth noting that BC extracts contain much more phenolic compounds than the two other plants, while HAW extracts contain much more proanthocyanidin oligomers than the two other plants. CA and BC extracts contain similar amounts of flavonoids. UHPLC revealed that the major compounds detected in UV are flavonol in BC; hydrocinnamic acid derivatives, flavone flavanone and aurone in CA; flavanol, flavonol, and flavone in HAW. UHPLC-ESI-MS profiles revealed the presence of Flavanomarein and Martitimein derivatives in CA extracts, while FT-ICR-MS revealed the specific presence of Oleanolic or Ursolic acid. In BC, Quercetin and Kaempferol derivatives were mainly identified. Vitexin-2-O-rhamnoside, Hyperoside and Isoquercetin were the main components in HAW. 

Regarding their enzymatic and antioxidant activities, a remarkable hyaluronidase inhibition, superior to 90%, was reported for HAW extracts (at 1 g L^−1^) similar to the inhibition observed with the referenced hyaluronidase inhibitor. The inhibition by BC and CA extracts was lower; 64% and 60%, respectively. As for the antihypertensive activity, CA extracts demonstrated the highest ACE inhibition (91–97% at 1 g L^−1^), followed by HAW extracts (81–92%); while BC extracts gave the lowest percentage of ACE inhibition (72–76%). About the antioxidant activity, BC extracts had the highest antioxidant capacity in correlation with the highest contents in TPC and TFC, while CA extracts presented the lowest contents in TPC and TFC and the lowest antioxidant capacity.

This work highlights the complexity in the composition of these plant extracts, either in number of chemical components or in the chemical structure. It also confirms the interest of these medicinal plants toward a broad range of biological activities. In the future, it seems promising to investigate biological/pharmacological activities that are different from the primary therapeutic intention. Their use as daily nutrient in food toward the prevention of chronicle disease would also be of interest.

## Figures and Tables

**Table 1:** List of samples (with lot numbers) studied in this work with the corresponding experimental investigation. For each lot number, three plant extract samples coming from independent extractions were tested. ^a^: on raw and ground 1 mm granulometry; ^b^: on raw, ground 1 mm and ground ‘fine’ granulometry; ^c^: on ground ‘fine’ granulometry; ^d^: all extraction modes were tested; ^e^: optimized infusion protocol only; ^f^: ground 1 mm granulometry. For the granulometry, see Section 2.2. All extraction modes are described in Section 2.3, Section 2.4, Section 2.5, Section 2.6, Section 2.7, Section 2.8

	Kinetics of Extraction ^a^	TPC ^b^, TFC ^b^, OPC ^b^	UHPLC-MS ^c^	ESI(-) FT-ICR-MS ^c^	Enzymatic Activity ^c^
*Chrysanthellum americanum* (CA)	559980 (infusion and maceration)	559980 ^d^	559980 ^e^, CP44120 ^e^, NH558088 ^e^	559980 ^e^, CP44120 ^e^, NH558088 ^e^	559980 ^e^
Blackcurrant (*Ribes nigrum*) leaves (BC)	55870 (infusion and maceration)	55870 ^d^	55870 ^e^, NH558024 ^e^	55870 ^e^	55870 ^e^
Hawthorn (*Crataegus*) flowering tops (HAW)	20335 (infusion, maceration and ultrasonic) [4]	20335 ^d^ [4]	20335 ^d^/R78927 ^e^, 1221478 ^e^, H18001534 ^e^, CB58120 ^e^ [4]	CB58120 ^e^, APC27031904 ^e^	20335 (infusion) ^f^ CB58120 ^e^

**Table 2:** Physicochemical characteristics of the extracts of CA and BC depending on the extraction time, the extraction mode, and the plant granulometry. A total of 2.5 g of plant material in 250 mL water was systematically used. In total, 100 µL of herbal tea solution was taken and added to 4 mL water before UV measurement, except for ^a^, where 100 µL was added to 8 mL water to avoid spectrometer saturation (reported values were then multiplied by 2 for pertinent comparison). ^b^: ±1 standard deviation (*n* = 3 repetitions). ^c^: in mg equation GA/g dry plant, ± one standard deviation (*n* = 3 repetitions). ^d^: in mg equation Q/g dry plant, ± 1 SD (*n* = 3 repetitions). ^e^: in mg equation CY/g dry plant, ±1 SD (*n* = 3 repetitions). Lot number for CA: 559980. Lot number for BC: 55870.

Plant Nature and Granulometry	Extraction Mode	Extraction Time to Get 70% of the Abs at 30 min (min)	Absorbance at 30 min	10 min Extraction Time				30 min Extraction Time			
				Extraction Yield (%) ^b^	TPC ^c^	TFC ^d^	OPC ^e^	Extraction Yield (%) ^b^	TPC ^c^	TFC ^d^	OPC ^e^
CA (raw)	Infusion	2	1.177	22.70 ± 0.94	11.29 ± 0.30	2.64 ± 0.06	0.36 ± 0.03	25.12 ± 1.96	14.79 ± 0.60	3.12 ± 0.07	0.43 ± 0.03
	Maceration	7	1.161	22.41 ± 0.45	8.37 ± 0.60	2.23 ± 0.18	0.34 ± 0.03	24.86 ± 1.48	11.48 ± 0.93	2.64 ± 0.22	0.37 ± 0.02
	Ultrasonic	-	-	24.12 ± 1.03	8.91 ± 0.44	3.21 ± 0.15	0.39 ± 0.03	-	-	-	-
	Microwave	-	-	27.61 ± 1.62	13.86 ± 0.60	3.29 ± 0.12	0.48 ± 0.02	-	-	-	-
	Percolation	-	-	16.60 ± 0.01	8.29 ± 0.05	2.08 ± 0.10	0.26 ± 0.02	-	-	-	-
CA (ground 1 mm)	Infusion	<1.5	1.647	31.81 ± 0.18	17.08 ± 0.31	4.42 ± 0.28	0.66 ± 0.04	32.34 ± 0.80	18.37 ± 0.51	3.62 ± 0.16	0.67 ± 0.06
	Maceration	<1.5	1.478	30.85 ± 0.41	12.87 ± 0.35	3.76 ± 0.05	0.50 ± 0.02	31.50 ± 0.47	14.59 ± 0.80	3.76 ± 0.16	0.54 ± 0.02
	Ultrasonic	-	-	31.65 ± 1.42	12.74 ± 0.94	4.21 ± 0.07	0.63 ± 0.05	-	-	-	-
	Microwave	-	-	31.29 ± 1.05	17.38 ± 0.83	4.21 ± 0.08	0.63 ± 0.02	-	-	-	-
	Percolation	-	-	31.09 ± 0.89	13.04 ± 0.48	4.32 ± 0.05	0.67 ± 0.04	-	-	-	-
BC (raw)	Infusion	8.5	1.824	15.16 ± 0.39	15.84 ± 0.45	1.72 ± 0.08	0.55 ± 0.04	20.69 ± 0.72	23.92 ± 1.38	2.48 ± 0.04	0.83 ± 0.07
	Maceration	15.5	1.137	8.44 ± 0.42	6.99 ± 0.35	0.81 ± 0.06	0.24 ± 0.02	18.49 ± 1.19	17.09 ± 0.28	1.98 ± 0.11	0.64 ± 0.01
	Ultrasonic	-	-	17.61 ± 1.48	18.82 ± 0.30	2.28 ± 0.17	1.02 ± 0.02	-	-	-	-
	Microwave	-	-	20.45 ± 0.21	23.08 ± 1.09	2.74 ± 0.21	1.02 ± 0.10	-	-	-	-
	Percolation	-	-	7.40 ± 0.38	6.50 ± 0.26	0.88 ± 0.01	0.30 ± 0.05	-	-	-	-
BC (ground 1 mm)	Infusion	<1.5	3.615 ^a^	30.49 ± 1.22	47.28 ± 0.57	4.75 ± 0.09	2.35 ± 0.17	32.41 ± 0.52	45.39 ± 1.25	4.82 ± 0.13	2.66 ± 0.16
	Maceration	<1.5	3.399 ^a^	30.39 ± 0.36	45.75 ± 2.34	4.73 ± 0.10	2.07 ± 0.05	30.51 ± 0.44	44.94 ± 1.35	4.70 ± 0.14	2.74 ± 0.16
	Ultrasonic	-	-	29.54 ± 0.35	42.42 ± 1.67	4.45 ± 0.32	2.55 ± 0.01	-	-	-	-
	Microwave	-	-	33.13 ± 0.27	50.62 ± 1.30	5.25 ± 0.09	3.60 ± 0.32	-	-	-	-
	Percolation	-	-	31.80 ± 0.96	46.05 ± 0.18	4.77 ± 0.01	2.42 ± 0.07	-	-	-	-

**Table 3:** Extraction yield, TFC, TPC, and OPC values in CA and BC extracts (‘fine’ ground) issued from infusion at 10 min extraction time using easy-to-use infusion extraction (2.5 g of plant material and 250 mL boiling water were introduced in Bodum^®^ French press recipient, see Section 2.8). Stirring was realized by manually rotating the recipient at the beginning of the infusion and 10 min later before filtration. ^a^: in mg equation. GA/g dry plant, ± 1 SD (*n* = 3 repetitions). ^b^: in mg equation Q/g dry plant, ± 1 SD (*n* = 3 repetitions). ^c^: in mg equation CY/g dry plant, ± 1 SD (*n* = 3 repetitions).

Nature of the Plant	Lot Number	Extraction Yield (%)	TPC ^a^	TFC ^b^	OPC ^c^
*Chrysanthellum americanum* (CA)	559980	25.9 ± 0.9	15.51 ± 0.05	3.31 ± 0.04	0.42 ± 0.01
Blackcurrant leaves (*Ribes nigrum*) (BC)	55870	28.6 ± 0.5	39.60 ± 0.49	4.44 ± 0.17	1.67 ± 0.10
Hawthorn (*Crataegus*) (HAW) [4]	CB58120	21.7 ± 0.1	20.1 ± 0.4	2.86 ± 0.02	1.81 ± 0.05

**Table 4:** Putative peak identification of the major compounds detected by UHPLC-DAD in the BC extracts. *λ*_max_ are the local maximum of absorbance on the UV spectrum. [M+H]^−^ and [M−H]^−^ columns provide the *m*/*z* value of the precursor ion. Other ions column gives the *m*/*z* value of the fragments that were detected in the MS spectra. Identification method using UV and ESI(+) and ESI(−) spectra. Lot number: 55870.

Peak	Retention Time (min)	*ƛ*_max_ (nm)	[M+H]^+^/[M−H]^−^	Other Ions in the Spectrum (Positive/Negative Mode)	Family (Subclass)	Identified Compound
1	3.25	219, 238, 325	355/353	377, 163/191, 179	Phenolic acid (hydroxycinnamic acid)	Chlorogenic acid
2	5.64	254, 353	611/609.2	303/301, 179	Flavonoid (Flavonol)	Quercetin 3-rutinoside
3^a^	5.75	254, 353	465/463.1	303/301	Flavonoid (Flavonol)	Quercetin 3-O-galactoside (hyperoside)
3^b^	5.85	254, 353	465/463.1	303/301	Flavonoid (Flavonol)	Quercetin 3-O-glucoside (isoquercetin)
4^a^	6.24	255, 353	551/549	303/505.1, 301	Flavonoid (Flavonol)	Quercetin-3-6-malonyl-glucoside
4^b^	6.24	263, 347	287/593	287/285	Flavonoid (Flavonol)	Kaempferol-3-O-rutinoside
5	6.51	265, 345	287/447.1	287/285	Flavonoid (Flavonol)	Kaempferol-3-O-hexoside
6	6.84	265, 344	535/533	287/489, 285	Flavonoid (Flavonol)	Kaempferol-malonylglucoside
7	7.05	265, 345	535/533	535, 287/489, 285	Flavonoid (Flavonol)	Kaempferol-malonyl-glucoside isomer

**Table 5:** Putatively peak identification of the major compounds detected by UHPLC-DAD in the CA extracts. *λ_max_* are the local maximum of absorbance on the UV spectrum. [M−H]^−^ column provides the *m*/*z* value of the precursor ion. Other ions column gives the *m*/*z* value of the fragments that were detected in the MS spectra. Identification method using UV and ESI (-) spectra. Lot number: 559980.

Peak	Retention Time (min)	*ƛ*_max_ (nm)	[M−H]^−^	Other Ions in the Spectrum	Family (Subclass)	Identified Compound
1	3.24	219, 238, 325	353	191, 179	Phenolic acid (Hydroxycinnamic acid)	Chlorogenic acid
8^a^	4.25	282	449	287, 151, 135	Flavonoid (Flavanone)	Eriodicyol-7-O-glucoside
8^b^	4.25	267	593	473, 353, 191	Flavonoid (Flavone)	6,8-C,C-diglucosylapigenin
9	5.51	284	449	287, 269, 151, 135	Flavonoid (Flavanone)	Isookanin-7-O-glucoside (Flavanomarein)
10	5.71	415	447	285, 151, 135, 133	Flavonoid (Aurone)	Maritimetin-6-O-glucoside (Maritimein)
11	5.83	280, 335	461	285	Flavonoid (Flavone)	Luteolin-7-O-glucuronide
12	6.24	208, 323	515	353, 191, 179, 173, 135	Phenolic acid (Hydroxycinnamic acid)	di-caffeoylquinic acid
13	6.45	211, 327	515	353, 191, 179, 173, 135	Phenolic acid (Hydroxycinnamic acid)	di-caffeoylquinic acid isomer
14	6.74	207, 266, 334	445	269	Flavonoid (Flavone)	Apigenin-7-glucuronide
15	6.90	209, 326	515	353, 191, 179, 173, 135	Phenolic acid (Hydroxycinnamic acid)	di-caffeoylquinic acid isomer

## Supplementary Materials

The following are available online at https://www.mdpi.com/2304-8158/9/10/1478/s1. Figure S1: Pictures of raw and ground CA and BC materials of various granulometries. Figure S2: MS spectra achieved by ESI(-) FT-ICR MS analysis of BC, CA and HAW with their corresponding heteroatom class distribution and van Krevelen diagram. Figure S3: Chemical structures of all compounds identified by UHPLC-ESI-MS. Figure S4: UHPLC profiles of two lots (55870 and NH558024) of BC infusion extracts obtained using Bodum^®^ recipient. Figure S5: UHPLC profiles of three lots (559980, CB44120 and NH558088) of CA infusion extracts obtained using Bodum^®^ recipient. Figure S6: Example of electropherograms showing the inhibitory effect of two extracts on hyaluronidase. Figure S7: Decreasing temperature profiles vs. time for different volumes of water in the Bodum^®^ container with or without added ice in the container. Table S1: Some species putatively assigned to m/z peaks observed specifically in BC, CA, and HAW samples. Table S2: Inhibition assays of hyaluronidase and ACE as well as the ABTS antioxidant capacity assay of the different plant extracts.
