# Supplementary material for: Water-Based Extraction of Bioactive Principles from Blackcurrant Leaves and Chrysanthellum americanum: A Comparative Study
Source: Foods. 2020 Oct 16;9(10):1478. doi: 10.3390/foods9101478 (PMC7602794; doi:10.3390/foods9101478)
Supplement: Supplementary file 1 [file foods-09-01478-s001.pdf]

SUPPORTING INFORMATION

Table of contents

Figure S1. Pictures of raw and grinded CA (top) and BC (bottom) materials of various granulometries. One small square on the pictures is 1 mm large (see also the scale on the picture). S1

Figure S2. Mass spectra achieved by ESI(-) FT-ICR MS analysis of BC (A), CA (B), and HAW (C) with their corresponding heteroatom class distribution and van Krevelen diagram.. .... S2

Figure S3. Chemical structures of all compounds identified by UHPLC-ESI-MS. S4

Figure S4. UHPLC profiles of two lots (55870 and NH558024) of BC infusion extracts obtained using Bodum® recipient.. S7

Figure S5. UHPLC profiles of three lots (559980, CB44120 and NH558088) of CA infusion extracts obtained using Bodum® recipient.. S7

Figure S6. Example of electropherograms showing the inhibitory effect of two extracts on hyaluronidase... S8

Figure S7. Decreasing temperature profiles vs time for different volumes of water (corresponding to a mug 250 mL or a bowl 405 mL) in the French-press Bodum® container with or without added ice in the container.. .... S9

Table S1. Some species putatively assigned to m/z peaks observed specifically in BC, CA and HAW samples but also commonly observed in all the samples analyzed by ESI(-) FT-ICR MS.. .... S10

Table S2. Inhibition assays of hyaluronidase and ACE as well as the ABTS antioxidant capacity assay of the different plant extracts. S14

Figure S1. *Pictures of raw and grinded CA (top) and BC (bottom) materials of various granulometries. One small square on the pictures is 1 mm large (see also the scale on the picture).*

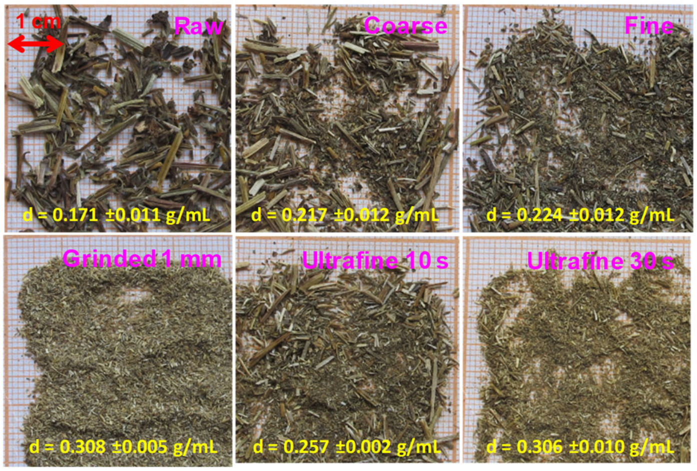

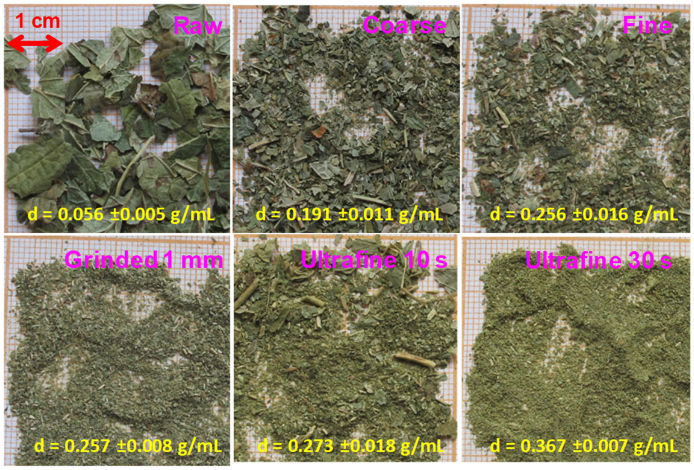

Figure S2. *Mass spectra achieved by ESI(-) FT-ICR MS analysis of BC (A), CA (B), and HAW (C) with their corresponding heteroatom class distribution and van Krevelen diagram. The size of the plots is relative to the peak intensity. See Table 1 for the list of all lot numbers investigated in this work.*

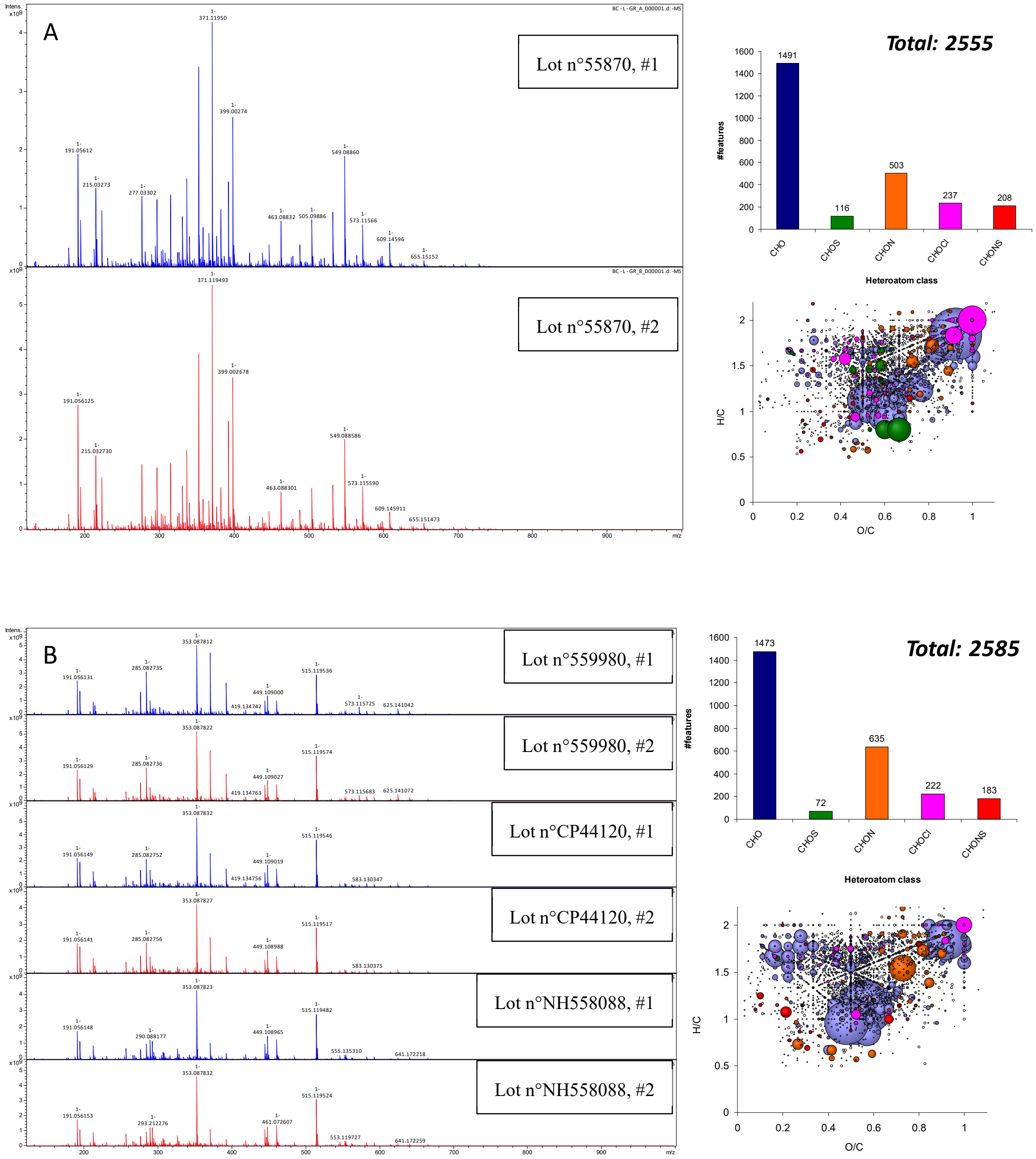
A
Lot n°55870, #1
Lot n°55870, #2
Total: 2555
1491
116
503
237
208
CHO
CHOS
CHON
CHOCl
CHONS
#features
Heteroatom class
H/C
O/C
B
Lot n°559980, #1
Lot n°559980, #2
Lot n°CP44120, #1
Lot n°CP44120, #2
Lot n°NH558088, #1
Lot n°NH558088, #2
Total: 2585
1473
72
635
222
183
CHO
CHOS
CHON
CHOCl
CHONS
#features
Heteroatom class
H/C
O/C
m/z

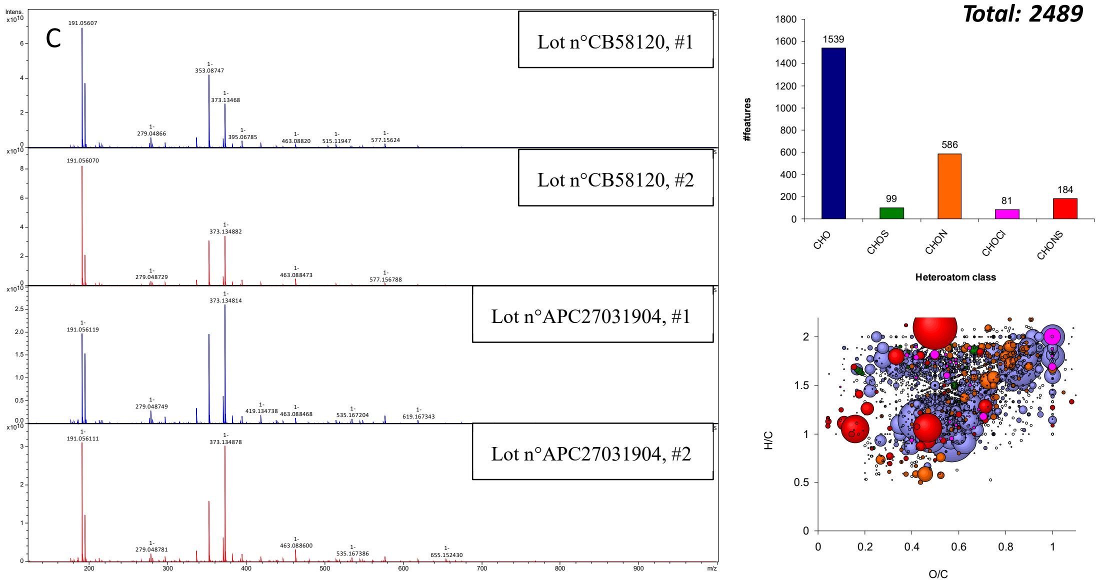

Figure S3. *Chemical structures of all compounds identified by UHPLC-ESI-MS.*

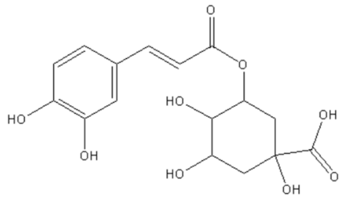	*$C_{16}H_{18}O_9$* *Exact mass: 354.0951*	*Peak 1: Chlorogenic acid*
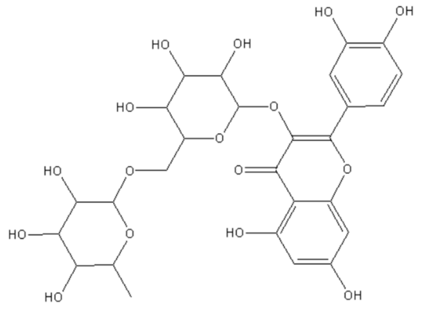	$C_{27}H_{30}O_{16}$ Exact mass: 610.1534	Peak 2: Quercetin-3-rutinoside
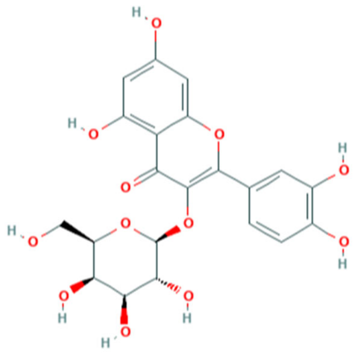	$C_{21}H_{20}O_{12}$ Exact mass: 464.0955	Peak 3[a]: Quercetin-3-O-galactoside (Hyperoside)

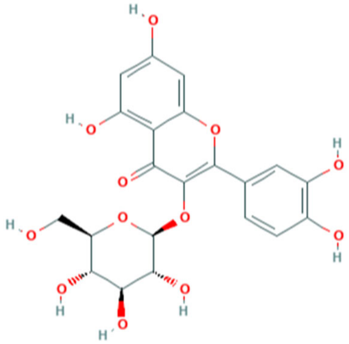	$C_{21}H_{20}O_{12}$ Exact mass: 464.0955	Peak 3[b]: Quercetin-3-O-glucoside (Isoquercetin)
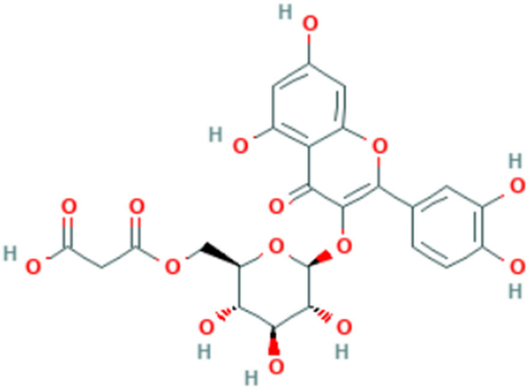	$C_{24}H_{22}O_{15}$ Exact mass: 550.0959	Peak 4[a]: Quercetin-3-6-O-malonyl-glucoside
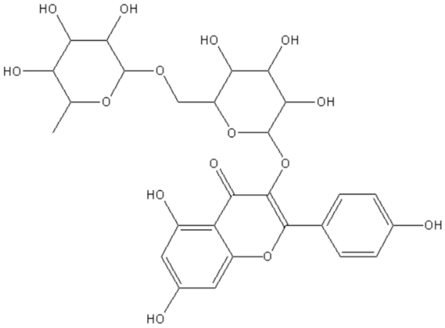	$C_{27}H_{30}O_{15}$ Exact mass: 594.1585	Peak 4[b]: Kaempferol-3-O-rutinoside
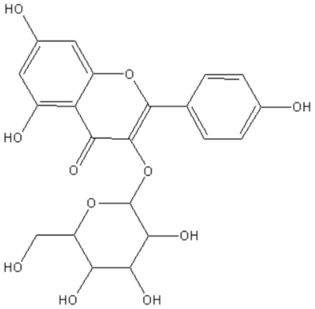	$C_{21}H_{20}O_{11}$ Exact mass: 448.1006	Peak 5: Kaempferol-3-O-hexoside
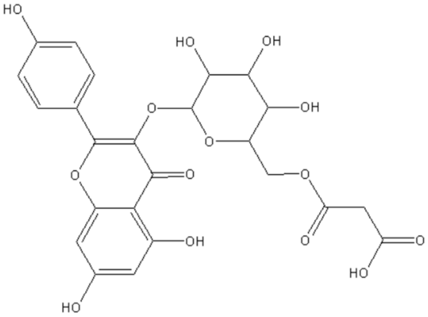	$C_{24}H_{22}O_{14}$ Exact mass: 534.101	Peak 6 and 7: Kaempferol-malonyl-glucoside and Kaempferol-malonyl-glucoside isomers

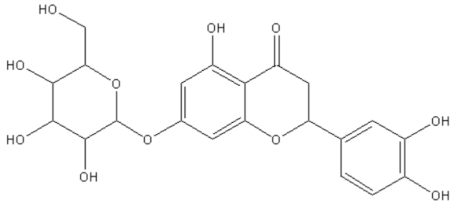	$C_{21}H_{22}O_{11}$ Exact mass: 450.1162	Peak 8[a]: Eriodicyol-7-O-glucoside
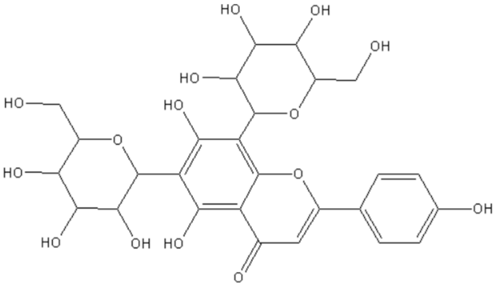	$C_{27}H_{30}O_{15}$ Exact mass: 594.1585	Peak 8[b]: 6,8-C,C-diglucosylapigenin
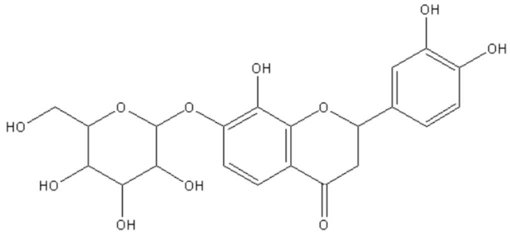	$C_{21}H_{22}O_{11}$ Exact mass: 450.1162	Peak 9: Isookanin-7-O-glucoside (Flavonomarein)
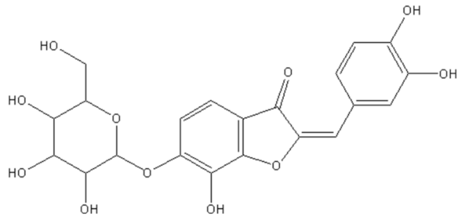	$C_{21}H_{20}O_{11}$ Exact mass: 448.1006	Peak 10: Maritimetin-6-O-glucoside (Martitimein)
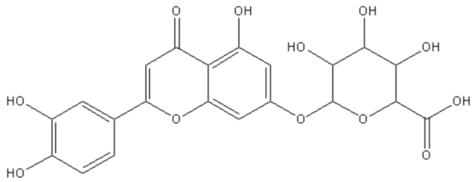	$C_{21}H_{18}O_{12}$ Exact mass: 462.0798	Peak 11: Luteolin-7-O-glucuronide
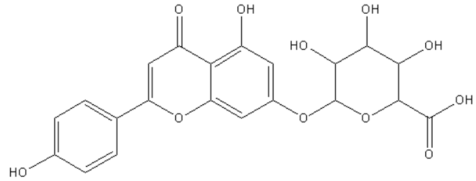	$C_{21}H_{18}O_{11}$ Exact mass: 446.0849	Peak 14: Apigenin-7-glucuronide

Peak 12, 13 and 15: three of six following isomers of di-caffeoylquinic acids

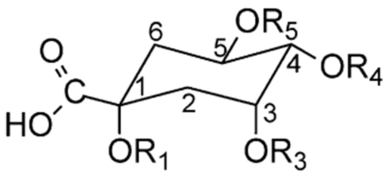

Quinic acid

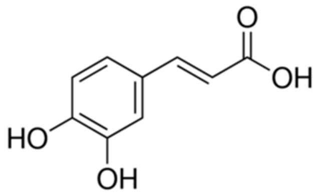

C = Caffeic acid

Name	**R_1**	**R_3**	**R_4**	**R_5**
1,3-di-O- caffeoylquinic acid	C	C	H	H
1,4-di-O- caffeoylquinic acid	C	H	C	H
1,5-di-O- caffeoylquinic acid	C	H	H	C
3,4-di-O- caffeoylquinic acid	H	C	C	H
3,5-di-O- caffeoylquinic acid	H	C	H	C
4,5-di-O- caffeoylquinic acid	H	H	C	C

Figure S4. *UHPLC profiles of two lots (55870 and NH558024) of BC infusion extracts obtained using Bodum® recipient.*

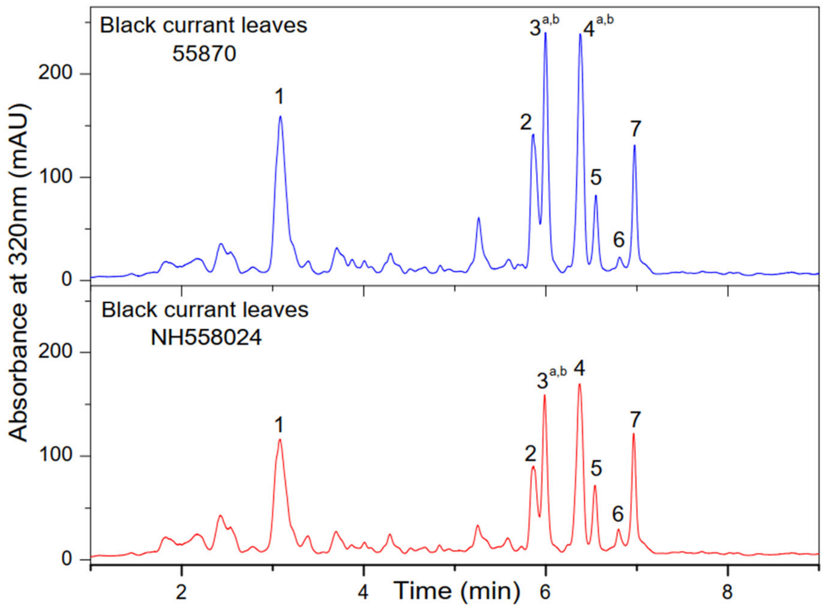

Figure S5. *UHPLC profiles of three lots (559980, CB44120 and NH558088) of CA infusion extracts obtained using Bodum® recipient.*

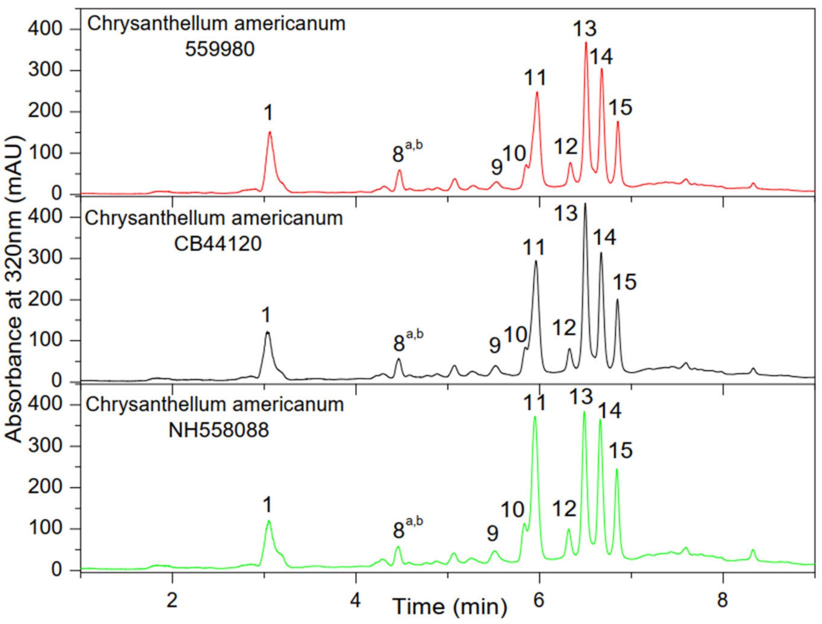

Figure S6. *Example of electropherograms showing the inhibitory effect of two extracts on hyaluronidase. EPG (1) enzymatic assay occurred normally in absence of plant extracts. Electropherograms number 2 and 4 correspond to raw aqueous extracts (b1 and f2, respectively) confirming the absence of interferents with tetrasaccharide (peak A), the final product of HA hydrolysis. Electropherograms number 3 and 5 are respectively the enzymatic reactions carried out in the presence of plant extracts b1 and f2. Reaction mixture in IB of control: 0.2 mg mL*$^{-1}$ *hyaluronidase and 0.8 mg mL*$^{-1}$ *HA. Modulation of hyaluronidase activity experimental conditions: 0.2 mg mL*$^{-1}$ *hyaluronidase, 0.8 mg mL*$^{-1}$ *HA and 1 mg mL*$^{-1}$ *of filtered raw extract. Incubation at 37 °C for 180 minutes. IB: 2 mM sodium acetate (pH 4.3). Electrophoretic separation conditions: BGE: 50 mM ammonium acetate (pH 8.9); anodic injection: 1.5 psi for 5 s; separation:+15 kV at 25°C; detection: λ = 200 nm; rinse between analyses at 30 psi: 5 min NaOH (1 M), 0.5 min water and 3 min BGE; bare-silica capillary: 57 cm total length, 47 cm detection length, 50 µm i.d. Peaks identification: electroosmotic flow (EOF,* t_m *= 7.3 min), peak A: tetrasaccharide(*t_m *= 13.5 min), peak B: hexasaccharide, peak C: octasaccharide, Hyaluronic acid (HA,* t_m*=17 min). All peaks were identified by CE-HRMS [1].*

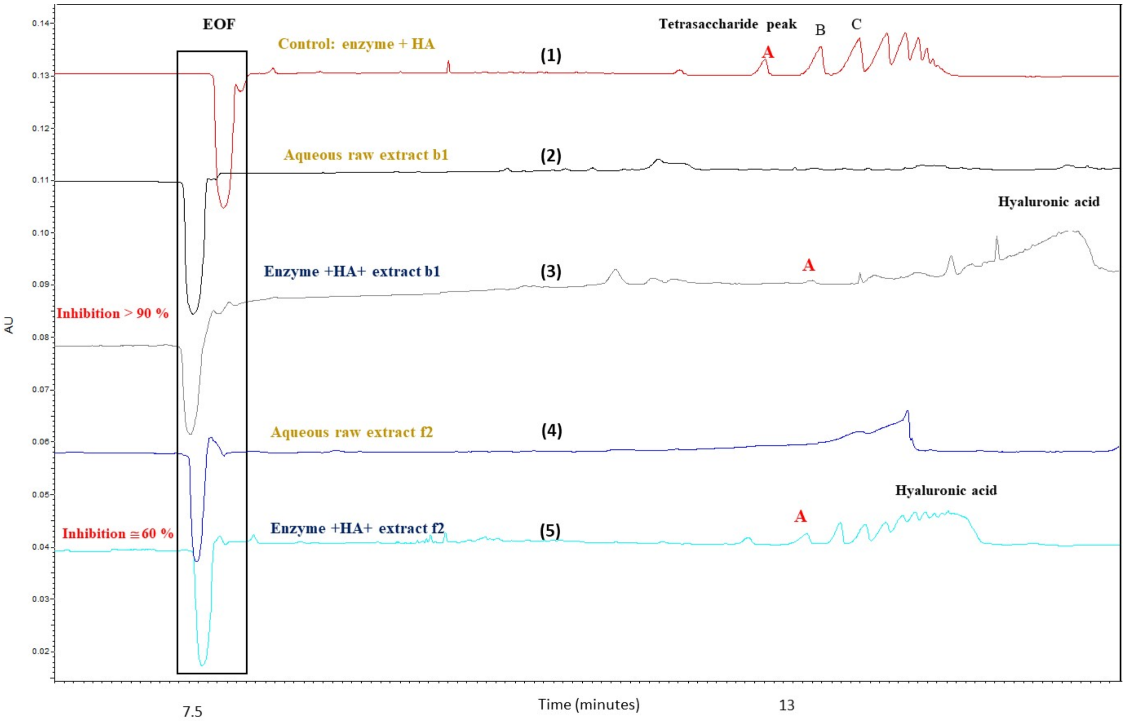

Figure S7. *Decreasing temperature profiles vs time for different volumes of water (corresponding to a mug 250 mL or a bowl 405 mL) in the French-press Bodum® container with or without added ice in the container. At each 3 min ± 10 s, 4 min ± 10 s, 5 min ± 10 s, one piece of ice (ice mass = 23.4 ± 1.5g) was added into the Bodum® container. Error bars are ± one SD on n=3 repetitions of independent extractions. The French-press Bodum® container was left open on the top and under stirring. Lines are only guides for the eyes.*

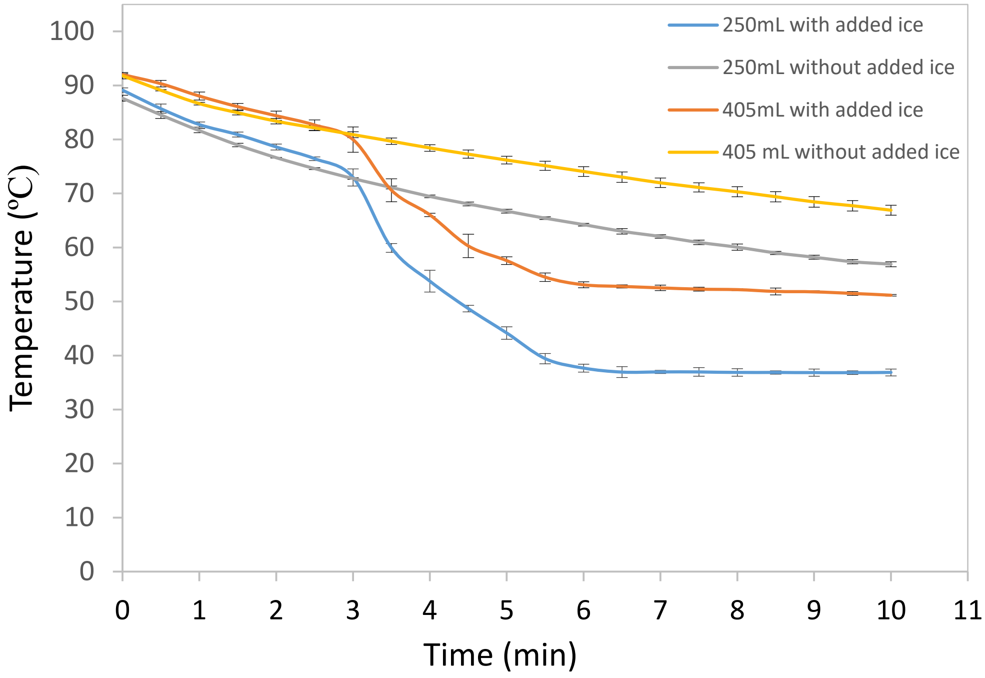

Table S1. *Some species putatively assigned to m/z peaks observed specifically in BC, CA and HAW samples but also commonly observed in all the samples analyzed by ESI(-) FT-ICR MS. Peaks of highest intensity are selected and putative assignment is based on identical molecular formulae achieved by ESI(-) FT-ICR MS and of identified compounds in Crataegus. Names in bold are species identified by UHPLC-DAD.*

	m/z	**Assignment**	**Candidate compound**	**Candidate compound**	**Category**	**Ref.**
**BC specific**	329.175898	C20H26O4	Carnosol	$[M-H]^-$	Diterpene	
	331.191497	C20H28O4	Carnosic acid	$[M-H]^-$	Diterpene	
	335.090313	C14H20O7	Salidroside	$[M+Cl]^-$		[2]
	341.043368	C15H14O7	(+)-Gallocatechin	$[M+Cl]^-$	Flavonoid	[3]
	343.082382	C18H16O7	Cirsilineol	$[M-H]^-$	Flavonoid	
	345.170802	C20H26O5	Rosmanol	$[M-H]^-$	Diterpene	
	347.075052	C11H20O10	Vicianose	$[M+Cl]^-$	Disaccharide	
	347.186439	C20H28O5	Gibberellin A53/A14/A15	$[M-H]^-$		[4]
	351.018013	C15H12O8S	5,7,4'-Trihydroxyflavanone 7-sulfate	$[M-H]^-$	Flavonoid	
	365.064481	C13H19O10P	Salicin 6-phosphate	$[M-H]^-$		
	383.163030	C20H28O5	Gibberellin A53/A14/A15	$[M+Cl]^-$		[4]
	399.157945	C20H28O6	Phorbol	$[M+Cl]^-$	Diterpene	
	589.192423	C29H34O13	Matteuorienate B	$[M-H]^-$	Flavonoid	
	601.119838	C28H26O15	(2S)-5,7,3',4'-Tetrahydroxyflavanone 7-(6-galloylglucoside)	$[M-H]^-$	Flavonoid	
	609.124844	C30H26O14	Gallocatechin-(4alpha->8)-epigallocatechin	$[M-H]^-$	Flavonoid	[5]
	629.127747	C27H30O15	Kaempferol 3-rhamnosyl-(1->2)-galactoside	$[M+Cl]^-$	Flavonoid	
	635.125281	C28H28O17	Acacetin 7-glucuronosyl-(1->2)-glucuronide	$[M-H]^-$	Flavonoid	
	645.101553	C30H26O14	Quercetin 3-(2''-p-coumarylglucoside)	$[M+Cl]^-$	Flavonoid	
	663.192874	C31H36O16	Pectolinarigenin 7-(4'''-acetylrutinoside)	$[M-H]^-$	Flavonoid	
	695.146424	C30H32O19	Quercetin 3-(6''-malonylneohesperidoside)	$[M-H]^-$	Flavonoid	
**CA specific**	231.06627	C13H12O4	Goniothalenol	$[M-H]^-$	Lactone	
	247.1339894	C15H20O3	Parthenolide	$[M-H]^-$	Sesquiterpene lactone	[6]
	251.1652585	C15H24O3	Indicumenone	$[M-H]^-$	Ketone	[7]
	274.129653	C12H21NO6	Glutarylcarnitine	$[M-H]^-$		
	289.104169	C11H18N2O7	N-Succinyl-LL-2,6-diaminoheptanedioate	$[M-H]^-$		

	317.066697	C16H14O7	Lecanoric acid	$[M-H]^-$	Polyphenol	
	327.051088	C17H12O7	(-)-Acanthocarpan	$[M-H]^-$	Flavonoid derivative	
	330.0322425	C12H13N3O4S	Acetylsulfamethoxazole	$[M+Cl]^-$		
	347.2439396	C18H36O6	Sativic acid	$[M-H]^-$	Fatty acid	
	371.1347112	C17H24O9	Syringin	$[M-H]^-$	Monosaccharide derivative	[8]
	371.2439485	C20H36O6	19(R)-hydroxy-Prostaglandin F1α	$[M-H]^-$	Fatty acid	
	433.0958854	C26H42O3S	1alpha,25-dihydroxy-3-deoxy-3-thiavitamin D3 3-oxide / 1alpha,25-dihydroxy-3-deoxy-3-thiacholecalciferol 3-oxide	$[M-H]^-$		
	455.353069	C30H48O3	Oleanolic/ursolic acid	$[M-H]^-$	Triterpene	[9–11]
	457.1140505	C21H20O10	Vitexin	$[M+Cl]^-$	Flavonoid	
	529.135034	C26H26O12	Luteolin 3'-methyl ether 7-(6''-crotonylglucoside)	$[M-H]^-$	Flavonoid	
	561.2397738	C35H34N2O5	Trilobine ([M-H]-)	$[M-H]^-$	Alkaloid	
	563.1767775	C27H32O13	Pinocembrin 7-rhamnosylglucoside	$[M-H]^-$	Flavonoid	
	609.2838795	C32H46O9	Cucurbitacin A	$[M+Cl]^-$	Cholesterol derivative	
	633.2245624	C24H42O19	Lactodifucotetraose	$[M-H]^-$	Polysaccharide	
	725.208519	C36H38O16	Licorice glycoside C1/C2	$[M-H]^-$	Flavonoid	
**HAW specific**	131.046229	C4H8O3N2	Asparagine	$[M-H]^-$	Amino acid	[12]
	193.035397	C6H10O7	Galacturonic acid	$[M-H]^-$	Saccharide derivative	[13]
	195.066306	C10H12O4	Homoveratric acid	$[M-H]^-$	Phenol	
	201.113254	C10H18O4	Decanedioic acid	$[M-H]^-$	Fatty acid	
	203.0826113	C11H12O2N2	Tryptophan	$[M-H]^-$	Amino acid	[12]
	207.066307	C11H12O4	Sinapaldehyde	$[M-H]^-$	Phenol	
	210.077208	C10H13NO4	3-Methoxytyrosine	$[M-H]^-$	Amino acid derivative	
	217.083013	C8H14N2O5	Glutamylalanine	$[M-H]^-$	Dipeptide	
	355.123586	C19H25ClO2	11 beta-Chloromethylestradiol	$[M+Cl]^-$	Cholesterol derivative	
	369.067285	C12H18O13	Digalacturonic acid	$[M-H]^-$	Disaccharide	[13]
	387.040652	C17H17ClO6	Griseofulvin	$[M+Cl]^-$	Dibenzofuran	
	409.023334	C17H14O10S	Quercetin 3,7-dimethyl ether 4'-sulfate	$[M-H]^-$	Flavonoid	
	473.072564	C22H18O12	Chicoric acid	$[M-H]^-$	Phenol	

515.226281	C21H41O12P	1-dodecanoyl-glycero-3-phospho-(1'-myo-inositol)	$[M-H]^-$	Monoacylglycero phosphoinositol
537.192084	C33H30O7	8-trans-[2-(6-Benzoyloxy-4-hydroxy-2-methoxy-3-methylphenyl)ethenyl]-5-methoxyflavan-7-ol	$[M-H]^-$	Flavonoid
613.132937	C27H30O14	Kaempferol 3-rhamnoside-(1->2)-rhamnoside	$[M+Cl]^-$	Flavonoid
663.156574	C30H32O17	Apigenin 7-(6''-malonylneohesperidoside)	$[M-H]^-$	Flavonoid
725.193469	C32H38O19	Schaftoside 6''-O-glucoside	$[M-H]^-$	Flavonoid
737.193737	C33H38O19	Kaempferol 7-methyl ether 3-[3-hydroxy-3-methylglutaryl-(1->6)]-[apiosyl-(1->2)-galactoside]	$[M-H]^-$	Flavonoid
769.198376	C37H38O18	Isovitexin 2''-O-(6'''-feruloyl)glucoside	$[M-H]^-$	Flavonoid

	m/z	Assignment	Candidate compound	Adduct	Category	Ref.
**Common features**	132.030259	C4H7O4N	Aspartatic acid	$[M-H]^-$	Amino acid	[9–11]
	133.014272	C4H6O5	Malic acid	$[M-H]^-$	Organic acid	[9–11]
	137.024450	C7H6O3	Protocatechuic aldehyde/hydroxybenzoic acid	$[M-H]^-$	Phenol	[9–11]
	146.045903	C5H9O4N	Glutamatic acid	$[M-H]^-$	Amino acid	[9–11]
	153.019355	C7H6O4	Protocatechuic acid	$[M-H]^-$	Phenol	[9–11]
	163.040092	C9H8O3	Coumaric acid	$[M-H]^-$	Coumaric acid	[9–11]
	179.035004	C9H8O4	Caffeic acid	$[M-H]^-$	Caffeic acid	[9–11]
	179.056137	C6H12O6	Glucose/fructose/Inositol	$[M-H]^-$	Monosaccharide	[9–11]
	181.071782	C6H14O6	Sorbitol	$[M-H]^-$	Monosaccharide	[9–11]
	188.035337	C10H7O3N	alpha-Cyano-4-hydroxycinnamic acid (HCCA)	$[M-H]^-$	Phenol	[9–11]
	191.019742	C6H8O7	Citric acid	$[M-H]^-$	Organic acid	[9–11]
	191.056085	C7H12O6	Quinic acid	$[M-H]^-$	Phenol	[9–11]
	193.050666	C10H10O4	Ferulic acid	$[M-H]^-$	Phenol	[9–11]
	223.061189	C11H12O5	Sinapinic acid	$[M-H]^-$	Phenol	[9–11]
	285.040487	C15H10O6	Kaempferol/Cyanidin (-2H-)	$[M-H]^-$	Flavonoid	[9–11]

289.071827	$C_{15}H_{14}O_6$	Catechin/epicatechin	$[M-H]^-$	Flavonoid	[9–11]
301.035432	$C_{15}H_{10}O_7$	Quercetin	$[M-H]^-$	Flavonoid	[9–11]
315.051075	$C_{16}H_{12}O_7$	Sexangularetin	$[M-H]^-$	Flavonoid	[9–11]
331.067100	$C_{13}H_{16}O_{10}$	Galloylglucose	$[M-H]^-$	Tannin	[9–11]
341.109006	$C_{12}H_{22}O_{11}$	Sucrose	$[M-H]^-$	Saccharide	[9–11]
353.087829	$C_{16}H_{18}O_9$	**Chlorogenic acid / 5-O-Caffeoylquinic acid**	$[M-H]^-$	Phenol derivative	[9–11]
417.082718	$C_{20}H_{18}O_{10}$	Kaempferol-O-arabinoside (crataegide)	$[M-H]^-$	Flavonoid	[9–11]
431.098376	$C_{21}H_{20}O_{10}$	Vitexin/Isovitexin/Apigenin-C-hexoside/Kaempferol O-pentoside	$[M-H]^-$	Flavonoid	[9–11]
447.093308	$C_{21}H_{20}O_{11}$	Orientin/Luteolin-C-hexoside/**Maritimetin-6-O-glucoside/Kaempferol-3-O-hexoside**	$[M-H]^-$	Flavonoid	[9–11]
449.108966	$C_{21}H_{22}O_{11}$	**Eriodictyol 7-O-glucoside/Isookanin-7-O-glucoside**	$[M-H]^-$	Flavonoid	[9–11]
461.072579	$C_{21}H_{18}O_{12}$	**Luteolin-7-O-glucuronide**	$[M-H]^-$	Flavonoid	[9–11]
461.108994	$C_{22}H_{22}O_{11}$	Methylluteolin-C-hexoside/Methoxykaempferol-pentoside	$[M-H]^-$	Flavonoid	[9–11]
463.088240	$C_{21}H_{20}O_{12}$	**Hyperoside/Isoquercitin**/Spiraeoside	$[M-H]^-$	Flavonoid	[9–11]
477.103886	$C_{22}H_{22}O_{12}$	Sexangularetin 3-O-glucoside	$[M-H]^-$	Flavonoid	[9–11]
489.103836	$C_{23}H_{22}O_{12}$	O-Acetylorientin	$[M-H]^-$	Flavonoid	[9–11]
505.098780	$C_{23}H_{22}O_{13}$	Quercetin-O-acetyl hexoside	$[M-H]^-$	Flavonoid	[9–11]
515.119522	$C_{25}H_{24}O_{12}$	**Dicaffeoylquinic acid**	$[M-H]^-$	Phenol	[9–11]
533.093590	$C_{24}H_{22}O_{14}$	**Kaempferol-malonylglucoside**	$[M-H]^-$	Flavonoid	[9–11]
563.104079	$C_{25}H_{24}O_{15}$	Sexangularetin 3-O-(malonyl)glucoside	$[M-H]^-$	Flavonoid	[9–11]
577.156128	$C_{27}H_{30}O_{14}$	Iso/vitexin rhamnoside	$[M-H]^-$	Flavonoid	[9–11]
593.151052	$C_{27}H_{30}O_{15}$	**Kaempferol-3-O-neohesperidoside**/Iso/Orientin O-rhamnoside	$[M-H]^-$	Flavonoid	[9–11]

609.145975	C27H30O16	**Rutin**/Quercetin-3-O-rhamnosylgalactoside/**Quercetin 3-rutinoside**	$[M-H]^-$	Flavonoid	[9–11]
623.161612	C28H32O16	Sexangularetin 3-neohesperidoside/Metoyxykaempferol methylpentosylhexoside	$[M-H]^-$	Flavonoid	[9–11]
625.140986	C27H30O17	**6,8-Diglucosylapigenin**	$[M-H]^-$	Flavonoid	[9–11]

Table S2. *Inhibition assays of hyaluronidase and ACE as well as the ABTS antioxidant capacity assay of the different plant extracts. For the enzymes inhibition assays, the plant extracts were screened at 1 mg mL^{-1} and the inhibition percentages of hyaluronidase and ACE were calculated according to Eq.2 and Eq.3, respectively. The antioxidant capacities of the plant extracts were determined at 0.01 mg mL^{-1} and calculated according to Eq.4. The absorbance of the multi-well plates was read twice for the ACE inhibition and ABTS antioxidant capacity assays. All assays were carried out in triplicates (n=3). Plant extracts were obtained from HAW (#1/#2, grinded 1 mm and #1/#2, grinded 'fine'), BC (#1/#2, grinded 'fine') and CA (#1/#2, grinded 'fine'). *EGCG, hyaluronidase referenced inhibitor, and Trolox, an antioxidant reference, were used to validate the methods. n.r.= Not relevant.*

	**Identification**	**Average hyaluronidase inhibition ± SD (%) ; n=3**	**Average ACE inhibition ± SD (%) ; n=3**		**Average reduction of ABTS absorbance ± SD (%) ; n=3 (Antioxidant Assay)**	
			**(Read #1)**	**(Read #2)**	**(Read #1)**	**(Read #2)**
Hyaluronidase Referenced inhibitor	EGCG*	100 %	n.r.	n.r.	n.r.	n.r.
Reference antioxidant compound	Trolox*	n.r.	n.r.	n.r.	64 ± 2	64 ± 2
Hawthorn	n°20335, #1	96 ± 1	89 ± 2	89 ± 2	45 ± 1	45 ± 1

flowering tops (HAW)	n°20335, #2	97 ± 2	81 ± 4	82 ± 4	45 ± 0	45 ± 0
	n°CB58120, #1	97 ± 1	91 ± 2	92 ± 2	34 ± 3	34 ± 3
	n°CB58120, #2	93 ± 1	88 ± 1	88 ± 1	28 ± 0	29 ± 0
Blackcurrant leaves (BC)	n°55870, #1	64 ± 2	76 ± 0	76 ± 0	58 ± 1	58 ± 1
	n°55870, #2	64 ± 3	73 ± 5	72 ± 5	64 ± 2	64 ± 2
Chrysanthellum americanum (CA)	n°559980, #1	61 ± 2	96 ± 2	97 ± 2	19 ± 2	19 ± 2
	n°559980, #2	59 ± 1	91 ± 2	91 ± 2	22 ± 0	22 ± 1

References

1. Fayad, S.; Nehmé, R.; Langmajerová, M.; Ayela, B.; Colas, C.; Maunit, B.; Jacquinet, J.C.; Vibert, A.; Lopin-Bon, C.; Zdeněk, G.; et al. Hyaluronidase reaction kinetics evaluated by capillary electrophoresis with UV and high-resolution mass spectrometry (HRMS) detection. *Anal. Chim. Acta* **2017**, *951*, 140–150.
2. Tung, Y.T.; Wu, M.F.; Lee, M.C.; Wu, J.H.; Huang, C.C.; Huang, W.C. Antifatigue activity and exercise performance of phenolic-rich extracts from calendula officinalis, ribes nigrum, and vaccinium myrtillus. *Nutrients* **2019**, *11*, 1715.
3. Karunaratne, D.N.; Pamunuwa, G. Food Addizives. In; BoD – Books on Demand, 2017.
4. Hess, D. Plant physiology: Molecular, biochemical, and physiological fundamentals of metabolism and development. In; Springer Science & Business Media, 2012.
5. Tits, M.; Angenot, L.; Poukens, P.; Warin, R.; Dierckxsens, Y. Prodelphinidins from Ribes nigrum. *Phytochemistry* **1992**, *31*, 971–973.
6. Blakeman, J.P.; Atkinson, P. Antimicrobial properties and possible rôle in host-pathogen interactions of parthenolide, a sesquiterpene lactone isolated from glands of Chrysanthemum parthenium. *Physiol. Plant Pathol.* **1979**, *15*, 183–190.
7. Mladenova, K.; Tsankova, E.; Kostova, I.; Stoianova-Ivanova, B. Indicumenone, A New Bisabolane Ketodiol from Chrysanthemum indicum. *Planta Med.* **1987**, *53*, 118–119.
8. Wei, Q.; Ji, X. ying; Long, X. shun; Li, Q. rong; Yin, H. Chemical Constituents from Leaves of "Chuju" Chrysanthemum morifolium and Their Antioxidant Activities in vitro. *Zhong yao cai* **2015**, *38*, 305–310.
9. Liu, P.; Yang, B.; Kallio, H. Characterization of phenolic compounds in Chinese hawthorn (Crataegus pinnatifida Bge. var. major) fruit by high performance liquid chromatography-electrospray ionization mass spectrometry. *Food Chem.* **2010**, *121*, 1188–1197.
10. Liu, P.; Kallio, H.; Yang, B. Phenolic compounds in hawthorn (Crataegus grayana) fruits and leaves and changes during fruit ripening. *J. Agric. Food Chem.* **2011**, *59*, 11141–11149.
11. Liu, P. Composition of hawthorn (Crataegus spp.) fruits and leaves and emblic leaf flower (Phyllanthus emblica) fruits, University of Turku, 2012.
12. McKenna, D.J.; Jones, K.; Hughes, K.; Tyler, V.M. *Botanical medicines: the desk reference for major herbal supplements*; Routledge, 2002;
13. Zhu, R.; Hong, M.; Zhuang, C.; Zhang, L.; Wang, C.; Liu, J.; Duan, Z.; Shang, F.; Hu, F.; Li, T.; et al. Pectin oligosaccharides from hawthorn (Crataegus pinnatifida Bunge. Var. major) inhibit the formation of advanced glycation end products in infant formula milk powder. *Food Funct.* **2019**, *10*, 8081–8093.
